# Benchmarking electronic-structure methods for nonadiabatic dynamics in a propeller-shaped molecular rotor: insights into conical intersections

**DOI:** 10.1039/d6fd00065g

**Published:** 2026-10-01

**Authors:** Marcus Brady, Rachel Crespo-Otero

**Affiliations:** a Department of Chemistry, University College London 20 Gordon Street WC1H 0AJ London UK r.crespo-otero@ucl.ac.uk

## Abstract

Understanding light-activated processes in molecular rotors is essential for applications ranging from energy storage to light-emitting materials. Rotation around single bonds provides an efficient nonradiative decay pathway, leading many molecular rotors to undergo quenching across environments. Nonadiabatic dynamics simulations offer a route to disentangling competing decay channels, but the predicted mechanisms depend sensitively on the level of theory. Here, we examine how different electronic-structure methods shape the topology of conical intersections and, in turn, the photochemical behaviour of *sym*-triphenylcyclopropene (Ph_3_C_3_H), a propeller-shaped rotor exhibiting low-quantum-yield emission from a locally excited state. Using exploratory-based surface-hopping dynamics with TD-CAM-B3LYP, ADC(2), and MRSF-TD-CAM-B3LYP, we characterise the S_2_–S_1_ and S_1_–S_0_ conical intersections and relate their topographies to the observed decay pathways. Consistent with the underlying potential-energy surfaces, the trajectories computed with ADC(2) evolve to S_0_ significantly faster than those obtained with TD-CAM-B3LYP and MRSF-TD-CAM-B3LYP. The S_2_–S_1_ intersection is peaked and bifurcating, promoting extensive population exchange, particularly for MRSF-TD-CAM-B3LYP. Two distinct S_1_–S_0_ intersections are identified: CI_1_, consistently peaked across CASSCF and all MRSF-TDDFT functionals, and CI_2_, which is sloped and single-path for all MRSF-TDDFT functionals but appears sloped and bifurcating at the MS-CASPT2 level. Across all methods, CI_1_ emerges as the dominant decay channel. MRSF-TD-CAM-B3LYP dynamics yield three outcomes: reformation of the initial geometry, formation of a diradical intermediate, and production of the bicyclic photoproduct, reflecting its bifurcating topology and more reliable ground-state description relative to LR-TDDFT. A systematic analysis of exchange–correlation functional dependence within MRSF-TDDFT reveals strong consistency across functionals, with only minor deviations such as the borderline single-path behaviour of BHHLYP and DTCAM-AEE. These findings raise important questions about the reliability, limitations, and future development of black-box excited-state methods for modelling photochemical decay in molecular rotors.

## Abstract 2

We examine how different electronic-structure methods shape the topology of conical intersections and, in turn, the photochemical behaviour of *sym*-triphenylcyclopropene (Ph_3_C_3_H).

## Graphical abstract

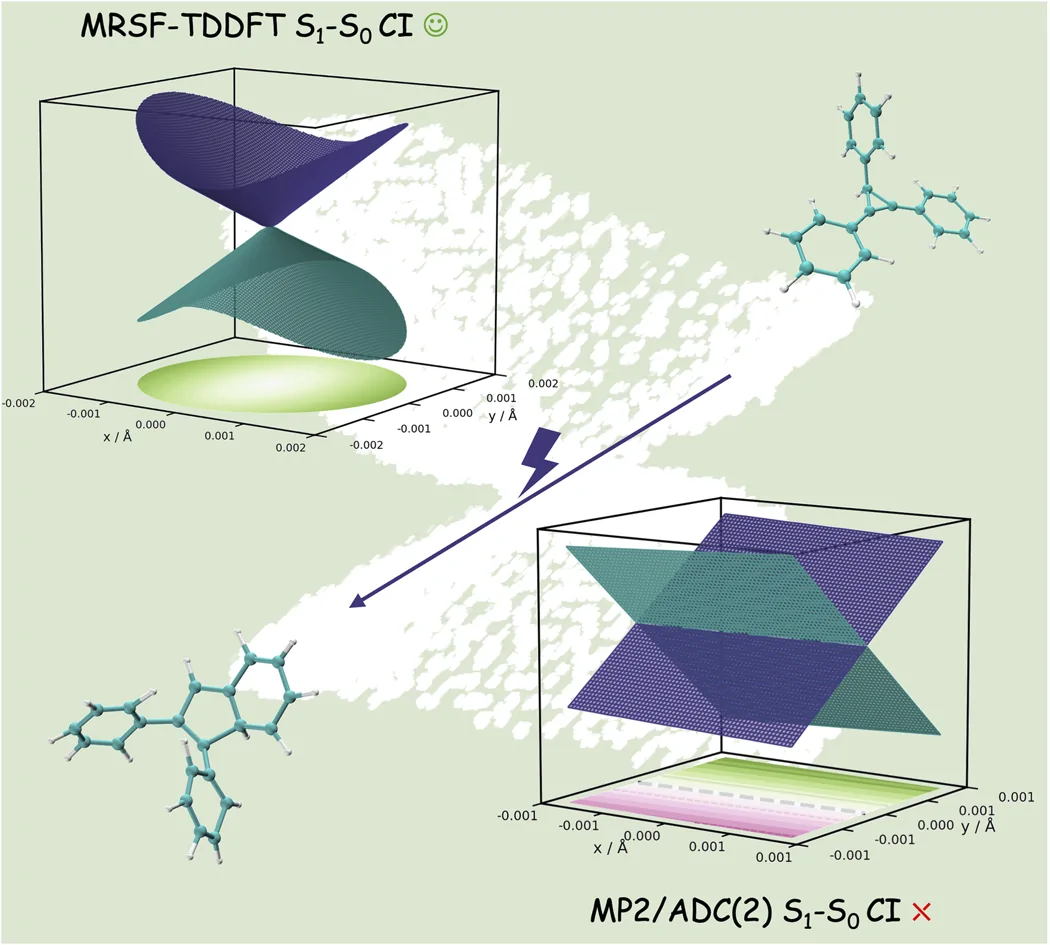

## 1 Introduction

Trajectory-based nonadiabatic dynamics allow the exploration of potential energy surfaces (PES) in excited states, providing insights into reaction pathways and quantum yields. Despite limitations due to its classical treatment of nuclear motion, surface hopping remains a widely used method due to its easy implementation and straightforward interpretation.^1^ However, the predictive power of nonadiabatic dynamics simulations is highly dependent on the accuracy of the underlying electronic structure methods. Inadequate electronic structure methods can lead to incorrect descriptions of the PES, impacting the reliability of the results.^2^ These issues motivated the recent prediction challenge on cyclobutanone dynamics, emphasising the need for more accurate electronic-structure methods for nonadiabatic simulations.^3^

The design of highly emissive materials in the condensed phase is of great interest for various applications but is limited by the presence of nonradiative decay pathways. As a result, materials exhibiting solid-state enhanced fluorescence have become a highly popular research topic.^4^ Many molecules that exhibit high emission in the solid state are molecular rotors with propeller-shaped structure, where the restricted solid environment limits vibrations that return the molecule to the ground state.^5–7^ Understanding the mechanisms behind quenching and how it can be modulated by the environment is crucial for developing more efficient materials. McGonigal *et al.* investigated a series of molecular rotors and analysed the effect of aromatic interactions on their emissive response in solution and the crystalline phase. The simplest system was *sym*-triphenylcyclopropene (Ph_3_C_3_H).^8^ In solution, Ph_3_C_3_H maintains a single emission peak, and they suggest it emits exclusively from its locally excited (LE) state rather than forming any intramolecular structures. Therefore, Ph_3_C_3_H (Fig. 1), like many other organic chromophores that are relevant for practical applications, is an aromatic system with possible multiconfigurational character and decay pathways through S_1_–S_0_ conical intersections. In previous work, we examined the excited-state dynamics of a propeller-based system and showed that, beyond rotation, ring puckering leading to a low-energy S_1_–S_0_ conical intersection plays a major role.^5^

**Fig. 1:**
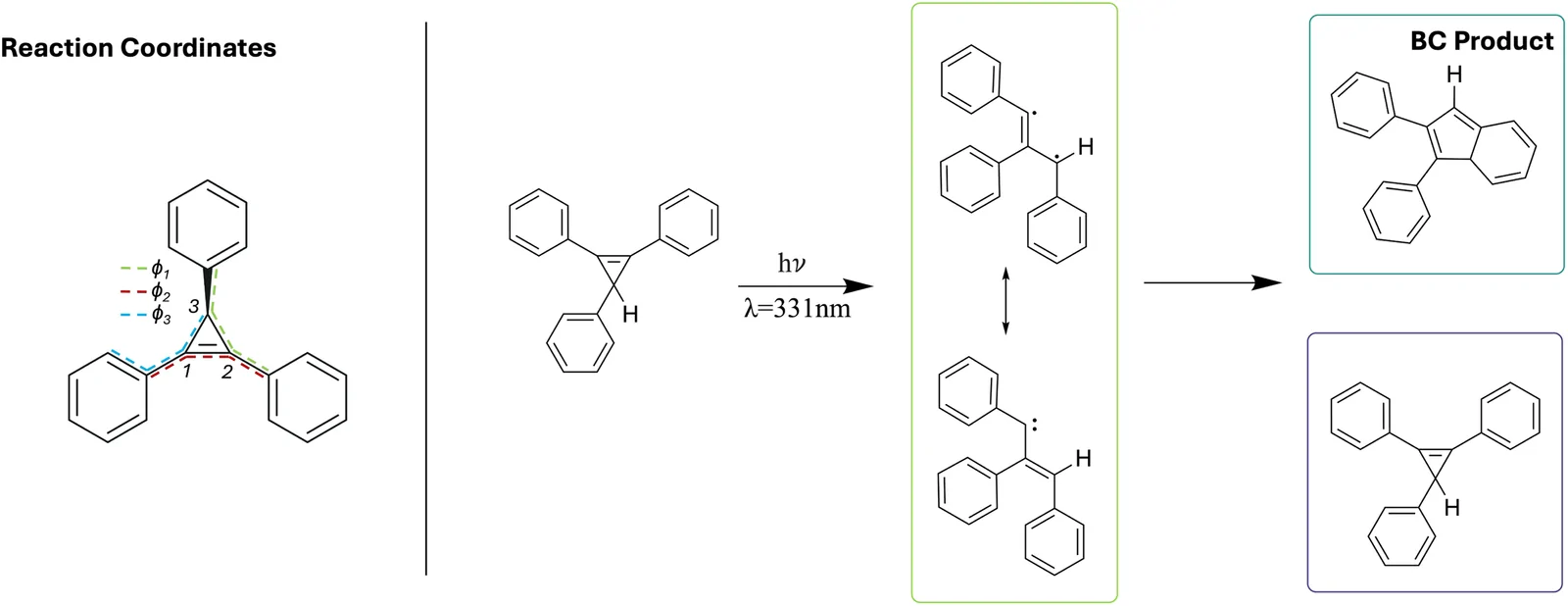
Left is the annotated ground state geometry with key reaction coordinates and right is the proposed reaction scheme; upon excitation, decay occurs through an S_1_/S_0_ conical intersection (in the green box) and then either reforms the starting reactant (purple box) or a bicyclic compound (cyan box, labelled BC product).

In this paper, we use surface hopping dynamics as an exploratory tool to identify the main radiative decay pathways in Ph_3_C_3_H, then we perform a thorough analysis of the S_2_–S_1_ and S_1_–S_0_ conical intersection topologies both visually and in terms of numerical characterisation.^9,10^ For the dynamics, we consider three different electronic structure methods: linear response (LR-)TDDFT, algebraic diagrammatic construction scheme to the second order (ADC(2)), and mixed reference spin-flip (MRSF-)TDDFT. LR-TDDFT tends to fail near intersections with the ground state due to the incorrect description of the topology of the intersection seam, and acts as two planes intersecting to form a seam instead of a conical intersection point.^1,11–14^ The calculations involving ADC(2) calculate the ground state through an MP2 calculation and the excited states, making a suitable description of CIs between excited states, but less so for those involving the MP2 ground state.^13,15^ The third method, MRSF-TDDFT, is built on the foundations of spin-flip (SF) TDDFT, which uses an unrestricted triplet ground state (*M*_s_ = +1).^1^ SF-TDDFT fixes some of the typical failing points of the LR-TDDFT, allowing double excitations and the description of conical intersections with the ground state but due to one triplet reference state it faces issues regarding spin-contamination.^11^ MRSF-TDDFT resolves this by using a reference ground state considering the two components of the triplet ground state (*M*_s_ = ±1),^16^ though still facing some issues due to the lack of some single excitations and instability of the triplet reference.^17^ Using this ensemble of electronic structure methods, we compare the quantitative and qualitative results of the corresponding nonadiabatic dynamics. For the analysis of the crossing seams, in addition to the methods used for the dynamics, additional XC functionals for MRSF-TDDFT are used for analysis of the ground state and first excited state conical intersections.

## 2 Computational details

For propagating the exploratory dynamics, we benchmark three different types of electronic structure methods: LR-TDDFT, MRSF-TDDFT, and ADC(2). The two different flavours of TDDFT both used the CAM-B3LYP functional with the def2-SVP basis set, since we later look at other XC functionals, these key ones we refer to as LR-TD-CAM-B3LYP and MRSF-TD-CAM-B3LYP. Since a large quantity of trajectories were propagated for a relatively large system (37 atoms), this was an adequate compromise for the level of theory needed. A benchmarking of the vertical excitations obtained with MRSF-TD-CAM-B3LYP and various basis sets can be found in the supplementary information (SI) in Table S1. Overall, the obtained vertical excitations show deviations on the order of 0.1 eV with respect to the larger basis set (cc-pVTZ), which seem to be reasonable. Calculations involving LR-TDDFT were performed in Gaussian 16,^18^ MRSF-TDDFT calculations utilised OpenQP.^16,19–21^ For the ADC(2) based calculations, the def2-SV(P) basis was used, all calculated using Turbomole and its RI-CC2 module.^22–25^ A subset of 20 trajectories was propagated using MRSF-TD-ωB97XD with the def2-SV(P) basis, starting from S_1_ and evolved for up to 2000 fs to explore the effects of different conical intersection seams. Two additional electronic structure methods were considered to provide higher level of theory reference, these were CASSCF (3-state and 6-state average) and MS-CASPT2 (3-state average) as implemented in OpenMolcas,^26^ these calculations used a symmetric active space of (12,12) and (10,10), respectively with the def2-SVP basis set for consistency. See Section S1 for the orbitals, used for all calculations.

The initial ground-state geometry of Ph_3_C_3_H was optimised using both DFT (in Gaussian 16) and MRSF-TDDFT (in OpenQP). The corresponding normal modes were computed, analytically for DFT and numerically for MRSF-TDDFT. Optimisations of other structures, including excited-state minima and the MRSF-TDDFT conical intersections, were carried out with OpenQP.^16,19–21^ To obtain the S_2_–S_1_ and S_1_–S_0_ MECIs with the LR-TDDFT and MP2/ADC(2) methods, we used the gas-phase conical-intersection search tool implemented in the fromage (FRamewOrk for Molecular AGgregate Excitations) software, employing geometries from the dynamics as starting points.^27–30^ The CASSCF and MS-PT2 conical intersections were obtained in OpenMolcas using the adaptive penalty technique.^26,31^ Wigner sampling the ground state minimum for 500 geometries provided the initial nuclear momenta and positions and hence the geometries for the absorption spectra in Fig. S3. We also include the spectrum as a decomposition of the individual excited states to see the S_3_ contribution (which is minimal), in Fig. S4. A subset of those were used for the initial conditions for dynamics. We initially started 50 trajectories in S_1_ and 20 in S_2_ for each electronic structure method. Due to the observed convergence issues in MRSF-TDDFT,^17,32–34^ the amount of trajectories starting in the S_1_ state was increased to 69.

The trajectories were propagated using the nonadiabatic mixed quantum–classical decoherence-corrected fewest-switches surface-hopping (DC-FSSH) algorithm in NewtonX,^35^ together with the corresponding interfaces to Gaussian 16 and Turbomole^2^ for LR-TD-CAM-B3LYP and ADC(2), respectively. The simplified decay of mixing (SDM) scheme was employed to correct decoherence issues.^36^ A recently developed interface between OpenQP and NewtonX was used to enable the calculation of MRSF-TD-CAM-B3LYP quantities for the dynamics.^32^ To propagate the electronic wavefunction, a time step of 0.025 fs was used, integrating the time-dependent Schrödinger equation with the Butcher fifth-order integrator.^37^ The nuclear degrees of freedom were propagated classically with a 0.5 fs time step using the velocity-Verlet algorithm. For consistency and to reduce computational cost, we used the energies and gradients from each electronic-structure method to compute the time-dependent Baeck-An (TD-BA) coupling as an approximation to the nonadiabatic couplings. After a hop, the velocities were rescaled along the momentum direction according to the full kinetic energy to determine whether a back-hop was allowed. It should be noted that there are reported issues when using TD-BA beyond a two-state system.^38^ Taking this into account, and since we observed hops between the S_1_ and S_2_ states during the simulations, we also performed a small test on a subset of LR-TD-CAM-B3LYP trajectories using the wavefunction-overlap method with local diabatisation to compute the nonadiabatic couplings,^35,39^ see Section S6.1.

Branching-plane grids around the conical intersections were generated using a new module added to fromage, taking the gradient difference and nonadiabatic coupling vectors (NACVs) as input; the relevant step sizes are provided in Section S4 in the SI. Gradient difference vectors were computed at each respective level of theory; all NACVs for ADC(2) could not be calculated and nor could the NACV calculated for the excited states of LR-TDDFT. As a substitute the MRSF-TDDFT quantities were used. For further reading, we refer the readers to ref. 40 and 41, as well as Section S4 in the SI for relevant derivations. Topological parameters 𝒫 and ℬ were calculated following ref. 9, 10, 15, 42 and 43 to classify each intersection as peaked/sloped and bifurcating/single-path. We provide further derivation in Section S5. The values used in the calculation of these parameters are also useful for classifying the conical intersections, where *σ* is the relative tilt parameter; measuring the magnitude of the energy gradient projected on to the branching plane vectors relative to the strength, *Δ*_gh_ measures the asymmetry of the conical intersection, and *θ*_s_ is the tilt of the CI.

## 3 Results

### 3.1 Static calculations

Table 1 shows the computed vertical excitations of Ph_3_C_3_H at both the Franck–Condon (FC) and the S_1_ minimum geometry. For the FC geometry, the lowest excited state (S_1_) is considered the bright state for all electronic structure methods used for dynamics (ADC(2), LR-TD-CAM-B3LYP, and MRSF-TD-CAM-B3LYP). There is a deviation between them of just over 0.3 eV for each. From the peak of the experimental absorption spectrum, we estimate the experimental S_0_ → S_1_ vertical excitation to be around 3.75 eV.^8^ Both LR-TD-CAM-B3LYP and MRSF-TD-CAM-B3LYP spectra (in Fig. S3) are in moderately good agreement with the experimental data, the ADC(2) spectrum shows a larger blue shift. Provided that we apply the shifts to account for deviations from the experimental spectrum, all obtained spectra can be used to generate the initial conditions, although LR-TD-CAM-B3LYP and MRSF-TD-CAM-B3LYP may offer a better description of the excited-state processes in Ph_3_C_3_H. The vertical excitations obtained with SA(3)-CASSCF[12,12]/def2-SVP and MS(3)-CASPT2[10,10] are 4.67 eV and 4.86 eV respectively, which are closer to the ADC(2) results with a larger deviation with respect to the experimental data, see Table S2. The experimental emission energy at low temperature (150 K) in 2-MeTHF is around 3.40 eV,^8^ and the values predicted with LR-TD-CAM-B3LYP and MRSF-TD-CAM-B3LYP are in very good agreement at the S_1_ minimum, whereas the ADC(2) value shows a larger blue shift (Table 1).

**Table 1:** Vertical excitation energies (eV) and the oscillator strengths (in brackets) from the S_0_ state at the FC and S_1_ minimum geometries. The basis set for all the calculations was def2-SVP

	Exp.	LR-TD-CAM-B3LYP	MRSF-TD-CAM-B3LYP	ADC (2)
**FC Geom**				
S_1_ (ππ*)	3.75	4.19 (0.79)	4.14 (1.31)	4.51 (0.97)
S_2_ (ππ*)	3.95	4.59 (0.03)	4.38 (0.03)	4.76 (0.02)
S_3_ (ππ*)	—	5.03 (0.00)	5.12 (0.00)	4.90 (0.00)

**S**_**1**_ **Min**.				
S_1_ (ππ*)	3.40	3.47 (0.78)	3.44 (1.31)	3.83 (1.02)
S_2_ (ππ*)	—	4.40 (0.03)	4.21 (0.03)	4.58 (0.01)
S_3_ (ππ*)	—	4.74 (0.00)	4.56 (0.00)	4.64 (0.02)

Fig. 1 shows the Franck–Condon geometry for Ph_3_C_3_H, the three central carbons on the cyclopropene ring are labelled for later analysis involving the reaction coordinates of the various bonds involving them (C_1_, C_2_, and C_3_). The schematic also shows the three important dihedral angles (*ϕ*_1_, *ϕ*_2_, and *ϕ*_3_) used to describe the rotational motion throughout dynamics. When one compares the differences between the FC and S_1_ minimum geometries, there is an increase in length of the double bond in the central ring (between C_1_ and C_2_). In turn, the two carbon single bonds (C_1_–C_3_ and C_2_–C_3_) become marginally shorter. See Section S8 in the SI for a quantitative comparison between S_0_ and S_1_ minimum geometries.

In our previous study of the cyclobutanone excited state dynamics, the role of higher lying electronic states has been questioned to impact the outcome of nonadiabatic dynamics,^32^ especially if contributions from those states are close to the excitation window in the simulated absorption spectra. For all three levels of theory, the contribution of S_3_ to the spectral region where the trajectories were generated was very small (0.2% for MRSF, 0% for TDDFT, and 0.8% for ADC(2)) (see Fig. S4).

### 3.2 Conical intersections, nonradiative decay pathways, and photoproducts

We identified two conical intersections relevant to the decay of Ph_3_C_3_H to S_0_ in the gas phase, CI_1_ and CI_2_. As will become clear later in this section, one of these plays a particularly significant role. We show both CI_1_ and CI_2_ in Fig. 2 as calculated and optimised for all the relevant levels of theory used for dynamics, we additionally include the RMSD with respect to the optimised MS-CASPT2 structure. The first decay pathway involving CI_1_ in Fig. 2 resulted from the breaking of one of the single C–C bonds in the central ring, C_1_–C_3_ or C_2_–C_3_. We provide a full comparison of the relative reaction coordinates of both CI_1_ and CI_2_ in the SI as well as a comparison of the RMSD values, Section S10. From this point, there were multiple pathways that the system could go through, as the dynamics was allowed to continue along the ground state for MRSF-TD-CAM-B3LYP we could also detect additional ground state processes at that level of theory. Following the wavepacket travelling through CI_1_, the system could remain as a diradical structure similar to that geometrically of the intersection geometry, shown by the product in the green box in Fig. 1. Most commonly following the hop to the ground state, the starting geometry was reformed (purple box in Fig. 1). Rarest of the photoproducts was the formation of a completely different product, resembling a [6,5] fused bicyclic compound with redistributed aromatic character (within the cyan box in Fig. 1 and we denote it as the BC product for brevity from here on out).

**Fig. 2:**
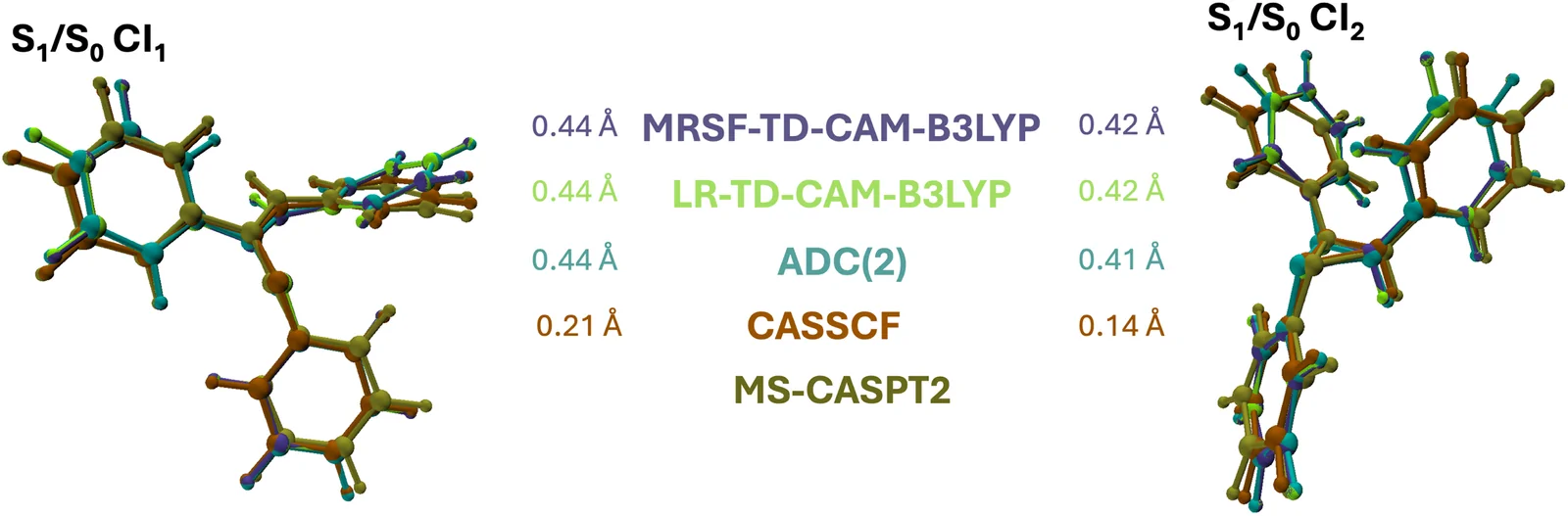
Two different conical intersection structures as found within the dynamics and thus optimised. Cyan for ADC(2) (with def2-SV(P)), lime for LR-TDDFT (TD-CAM-B3LYP/def2-SVP), purple for MRSF-TDDFT (TD-CAM-B3LYP/def2-SVP), ochre for CASSCF and dark green for MS-CASPT2. The RMSD (in Å) with respect to the MS-CASPT2 geometries are also included (see Section S10).

Initially, the unusual nature of the BC product led us to treat it with caution. However, a review of the literature revealed that it had been reported previously.^44–46^ Indeed, under both photochemical,^44^ and thermal conditions,^45^ homolytic cleavage commonly yields a diradical intermediate, closely resembling CI_1_. For the LR-TDDFT and ADC(2) dynamics, this geometry was a common hopping geometry that was optimised to very good agreement between all methods. Naturally, stopping the dynamics around the hops led to the lack of a photoproduct as a result from dynamics, instead optimising the hopping geometries to their respective ground states most commonly yielded the reformation of the starting product. We note these single reference methods did not form the BC product, however the BC product can be optimised and it is stable at the CAM-B3LYP/def2-SVP level of theory. The second CI structure (CI_2_ in Fig. 2) was obtained exclusively from dynamics using ADC(2) and was not found in either LR- or MRSF-TD-CAM-B3LYP dynamics. Here, the C=C double bond, between C_1_ and C_2_, extends and the respective phenyl groups are flipped into an up–down configuration. The reason for the absence of this hopping geometry for LR- and MRSF-TD-CAM-B3LYP dynamics is later discussed. To consider the possible energy surfaces and accessibility from the excited state of the FC geometry to the ground state involving these conical intersections, we geodesically interpolated^47^ from S_0_ → CI_1/2_ using the various electronic structure methods and the results can be seen in Fig. 3.

**Fig. 3:**
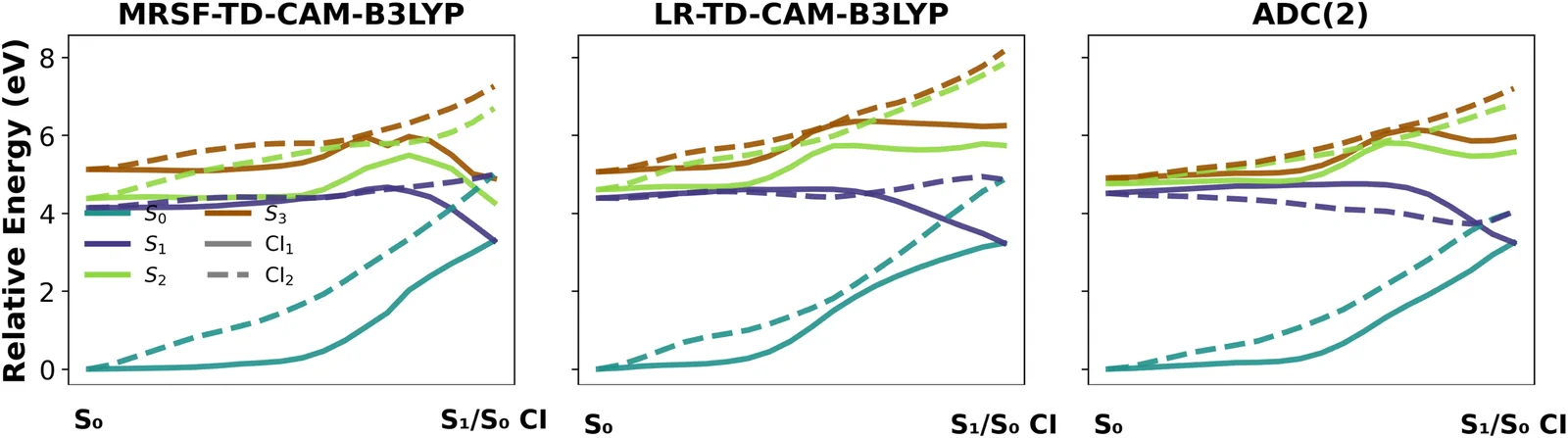
The potential energy surface for CI_1_ (solid) and CI_2_ (dashed) at three levels of theory: ADC(2) (with def2-SV(P)), LR-TDDFT (TD-CAM-B3LYP/def2-SVP), and MRSF-TDDFT (TD-CAM-B3LYP/def2-SVP). We include the ground state and the first three excited states.

Comparing the different pathways yields a good understanding of the particular roles of each conical intersection. This description is solely about the potential energy surface. Starting off with the first conical intersection we find it accessible, below the S_1_ energy at the Franck–Condon geometry, for all levels of theory hence its commonness within the dynamics. The gradients at which this point is reached varies between the methods, shallowest for LR-TD-CAM-B3LYP and more steep for both ADC(2) and MRSF-TD-CAM-B3LYP, which in turn have a slight energy barrier. This increased gradient towards the CI_1_ results in a higher momentum as the crossing is reached, directing it and making it an efficient transfer.^48^ One thing to note is the proximity of the S_2_ energy to the S_1_ energy midway through the surface, this is pertinent to all levels of theory. We identified an S_2_–S_1_ conical intersection that was frequently accessed during the dynamics, enabling population transfer from the initially excited S_1_ state to S_2_. This is most likely due to its geometry being similar to that of the starting Franck–Condon region. The corresponding geometries are shown in Fig. S6 in the SI, and the topology of these seams is discussed later in Section 3.4.1.

Moving to CI_2_ (dashed surface in Fig. 3), we can immediately rationalise why this conical intersection was found within ADC(2) dynamics, due to the ease of accessibility compared to the steep uphill pathway the wavepacket would be required to travel for both LR- and MRSF-TDDFT. In general, we can assume the topology of the surfaces for CI_2_ suggests it is characteristic of a sloped conical intersection. Indeed, whilst the potential energy surface is informative it is a singular dimensional perspective of the decay pathways, we hence perform a more thorough analysis of the topologies of the conical intersections later (Section 3.4). To assess the reliability of the MP2/ADC(2) calculations, we consider the *D*_1_ diagnostic.^49^ Although no benchmarks are available for this type of system, the Ph_3_C_3_H Franck–Condon geometry has *D*_1_ = 0.03, whereas CI_1_ and CI_2_ reach 0.08 along the interpolated potential energy surface, decreasing to 0.06 at the CI structure. This trend highlights the increasing difficulty of describing these regions of the PES and the need for multi-reference methods. We also note, the inclusion of the S_3_ state along the interpolated pathway in Fig. 3, showing the inaccessibility from the S_1_ state for both LR-TD-CAM-B3LYP and MRSF-TD-CAM-B3LYP. In the case of ADC(2), S_2_ and S_3_ remain very close in energy along the potential energy surface throughout the reaction coordinate. Since S_2_ depopulates very rapidly (reaching a population below 0.1 within 70 fs, Fig. 4) and both states separate relatively quickly from S_1_, their influence is expected to be minimal.

**Fig. 4:**
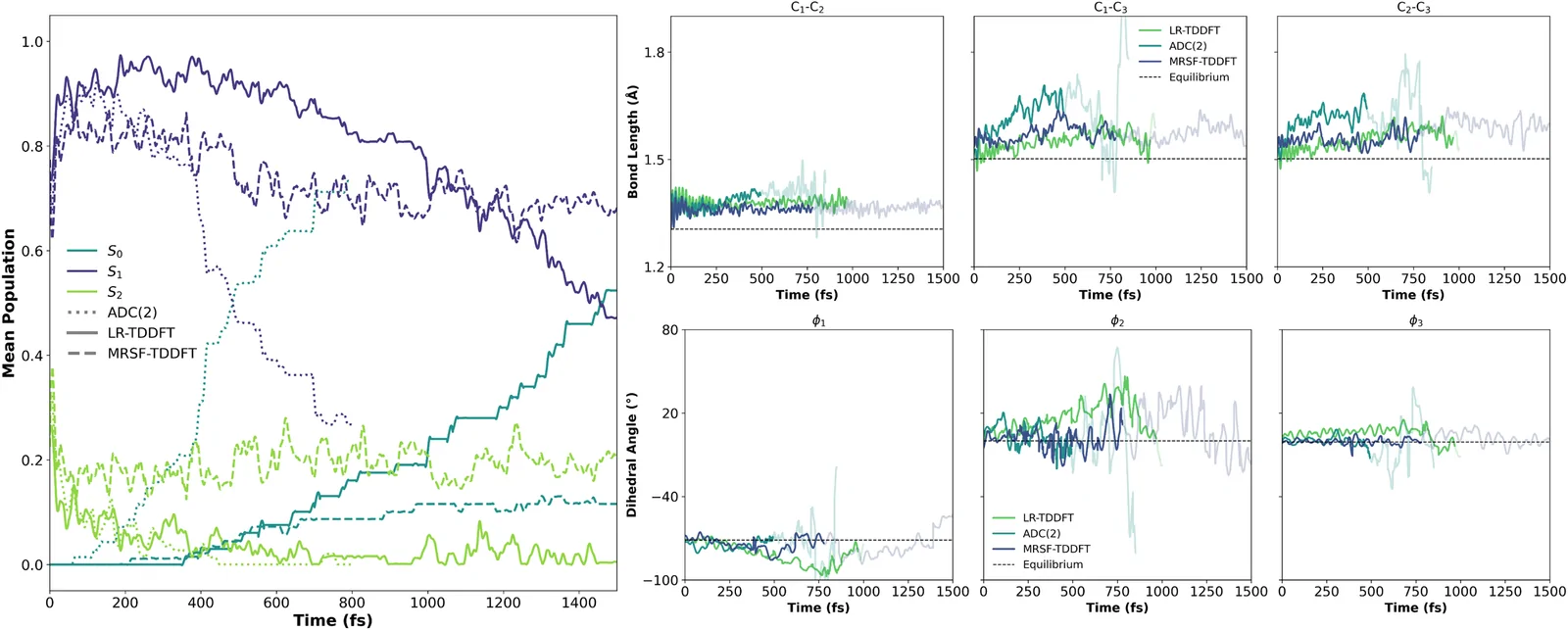
Time dependent populations and relevant geometrical parameters (Fig. 1) averaged over the trajectories, depending on the electronic structure used. Only the MRSF-TDDFT initial conditions based dynamics are considered for MRSF-TDDFT populations.

### 3.3 Nonadiabatic dynamics and a mechanistic analysis

We now move our attention to the analysis on the nonadiabatic dynamics. First, taking the trajectories that fell within the Δ*E*_S_1_–S_0__ < 0.2 eV condition, for the MP2/ADC(2) and LR-TD-CAM-B3LYP, their hopping geometries were optimised to get the aforementioned conical intersections. Dynamics for these levels of theory were halted here. Due to the satisfactory description of the ground state with MRSF-TDDFT we could allow hops to the ground state to occur, and in turn, keep running. This allowed for the investigation of the potential behaviour following a CI between the S_1_ and S_0_ states and hence the formation of various photoproducts (Section 3.2).

There was significant hopping activity between the S_2_ and S_1_ surfaces for MRSF-TD-CAM-B3LYP, more so than that for both LR-TD-CAM-B3LYP and ADC(2). An average trajectory, starting in S_1_, would hop 5.73 times between S_2_ ↔ S_1_ when using MRSF-TDDFT compared to 3.40 and 2.28 for LR-TDDFT and ADC(2), respectively, for the first 500 fs. To check this was not an artefact due to use of the TD-BA for the couplings, we took a minimal subset of trajectories and propagated dynamics with local diabatisation through wavefunction overlap. The corresponding trajectories behave similarly to those obtained using TD-BA, indicating that the hops to S_2_ arise from the small energy gap between the states along the reaction coordinate (3) and the presence of the S_2_–S_1_ conical intersection discussed further below (see Section S6.2 in the SI for the data and related plots). Some of the MRSF-TD-CAM-B3LYP trajectories experienced convergence issues due to the sensitivity and small changes to the restricted open-shell Hartree–Fock (ROHF) wavefunction. This has been observed in other systems, sometimes more severely.^17,32–34^ Since this is a relatively new method, issues like this are still yet to be addressed and for awareness of these issues collaboration between the nonadiabatic dynamics and electronic structure communities is recommended.

Fig. 4 shows the combined populations for all electronic structure methods, up to 1500 fs. It is clear upon first visual inspection that the quantitative results of the populations are very dependent on the electronic structure method. ADC(2) produces the faster dynamics, comparatively, with the depopulation to the ground state starting under 150 fs. The population transfer to the energetically higher S_2_ state is also fast and not long lived for ADC(2). Here, compared to LR-TD-CAM-B3LYP and MRSF-TD-CAM-B3LYP, ADC(2) was shown to have two different types of S_1_/S_0_ CIs allowing an increased number of decay pathways to the ground state. Additionally, the accessibility of the CI_2_ found in the potential energy surface for ADC(2) (Fig. 3a) could result in a higher chance of hopping to the ground state. For the LR-flavour of TD-CAM-B3LYP, it is much the same description of the ADC(2) dynamics but just on a slower scale, most likely by the harder to access ground state. MRSF-TD-CAM-B3LYP on the other hand shows a different picture. Initially, the S_1_ quite rapidly depopulates to the S_2_ state but unlike for the other electronic structure methods, the S_2_ population remains constant. This is largely due to a presence of an S_2_/S_1_ conical intersection as found in the potential energy surface (Fig. 3) that traps the wavepacket. We optimised the S_2_/S_1_ CI (seen in Fig. S6), and found it to be geometrically similar to the Franck–Condon geometry. This could in turn explain why an excess of trajectories ended in S_2_. We discuss the role of the S_2_/S_1_ CI topologies later in Section 3.4.1 to determine if this plays a significant role in the amount of time spent in S_2_ for the different electronic structure methods. To confirm electronic population convergence, we perform a convergence test and mean absolute deviation from the full set of trajectories in Section S14.

The averages for the geometrical parameters obtained with the electronic structure methods used in dynamics were analysed, the right of Fig. 4. Note, when there is a lower than half quantity of trajectories in the average, the line decreases in opacity. We start this analysis by first looking at the average different bond lengths. The standard oscillation of the C_1_=C_2_ double bond between 1.2–1.5 Å is common throughout all methods, this is expected considering, no matter the decay pathway, no dissociation occurs here. This is not the same case for the two single C–C bonds (C_1_–C_3_ and C_2_–C_3_), this requires to be broken to form CI_1_. The stretching of these bonds can be seen for all methods, particularly for ADC(2) when the sample size reduces the average becomes more irrational. As expected, the overall lack of a large deviation for these bond distances strongly correlates with the most common outcome: the reformation of the Franck–Condon geometry.

The CI_2_ found exclusively with ADC(2) results from the rotation of the phenyl groups about the central C=C bond to form the *trans*-like isomer of the starting product. This is where the use of the various dihedral angles becomes a useful metric. Given this rotation of the phenyl groups gives rise to CI_2_, specifically shown through the dihedral angle *ϕ*_2_, it would be rational for one to question why this is not present for LR-TD-CAM-B3LYP (lime) given the larger fluctuations from the equilibrium on average in the right of Fig. 4. In turn, why the ADC(2) (cyan) does not show this fluctuation yet the presence of CI_2_. Given the symmetry of the molecule there is the chance for the left phenyl group to flip up and the right to flip down and *vice versa*. When considering the individual trajectories for ADC(2) that decay through CI_2_, 20 form one configuration and 19 the other, resulting in an average near the equilibrium. This can be more fairly seen in the plot of individual trajectory reaction coordinates in Section S12.

In general, MRSF-TD-CAM-B3LYP is consistently near enough to the equilibrium values whilst weighted as more than half the trajectories are still running. Whilst the ensemble averages reflect the outcome of the reformation of the FC geometry, individual trajectories deviating from this provide an interesting geometric insight. Here we focus on two examples from MRSF-TD-CAM-B3LYP dynamics in Fig. 5, the first being the formation of the bicyclic product (compound in the cyan box in Fig. 1) and the second for the reformation of the Franck–Condon geometry. For these plots, we have the energy evolution over time on the left and the reaction coordinates on the right.

**Fig. 5:**
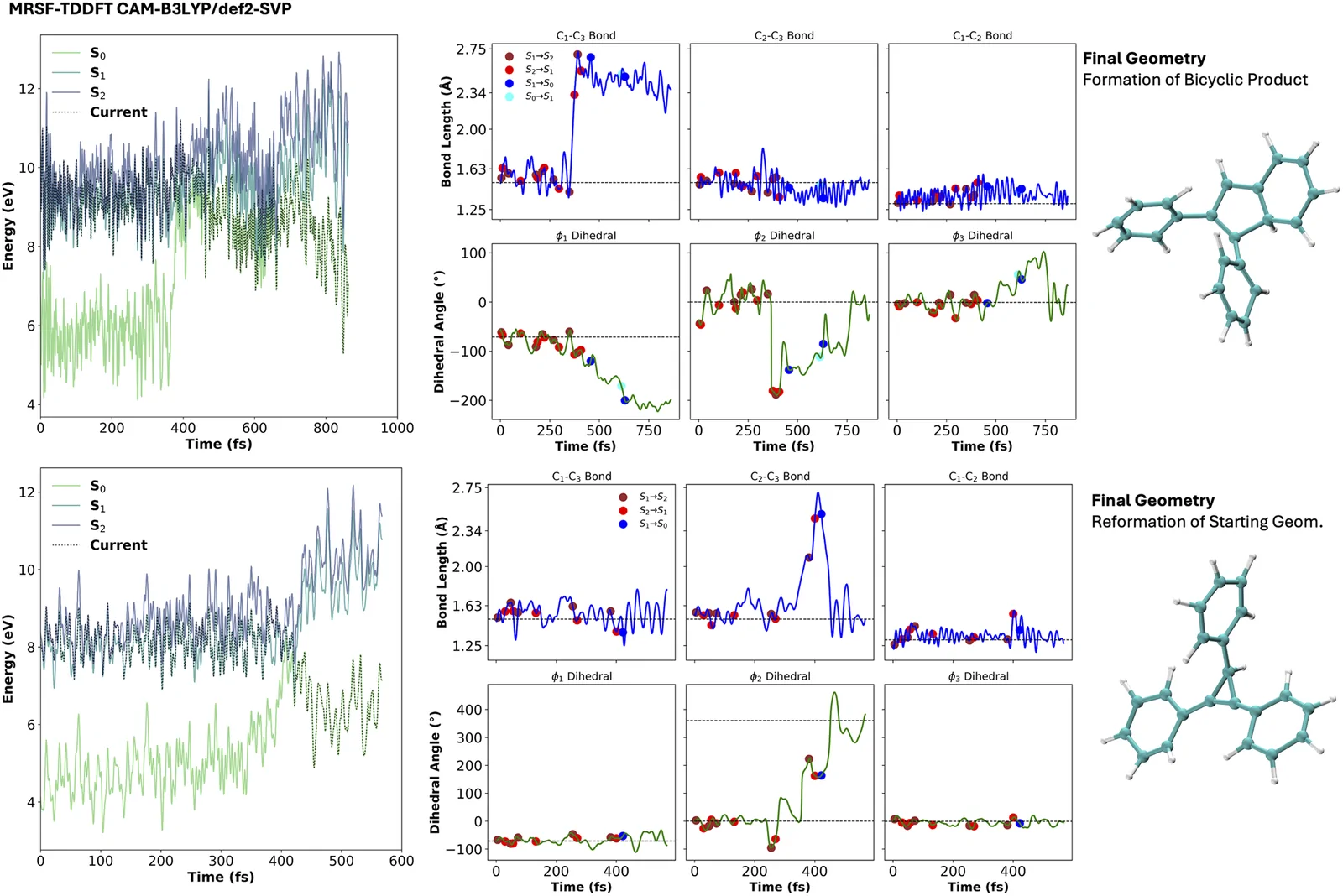
Two example trajectories from MRSF-TD-CAM-B3LYP, the evolution of the energies over time is on the left and the reaction coordinates are on the right. The hops between the electronic states are annotated.

For the formation of the bicyclic geometry, both *ϕ*_1_ and *ϕ*_2_ must rapidly change after the C_1_–C_3_ bond dissociation. This allows for the diradical intermediate to brought close enough to the phenyl group opposite the cyclopropene double bond. It appears *ϕ*_2_ equilibrates but it necessitates *ϕ*_1_ to stay high. The reformation of the starting geometry on the other shows a quick decrease in the C_2_–C_3_ bond length after the first dissociation, all whilst in the ground state. This is the main driving reaction coordinate for this scenario. In Fig. S8, we include an ADC(2) trajectory of similar format involving the hop to the ground state through CI_2_. At this point, it is important we iterate this is all gas phase behaviour and does not inherently translate to what would occur in the condensed phase, especially considering when aggregated, there would be π stacking of the coplanar phenyl rings. We would like to highlight that, although the description in the condensed phase is more complex, the analysis of the intramolecular nonradiative pathways remains relevant in more complex environments.

### 3.4 Conical intersection topologies

#### 3.4.1 S_2_–S_1_ conical intersection topology

Before visiting the S_1_/S_0_ conical intersection topologies we go over those calculated for S_2_/S_1_, the parameters (𝒫 and ℬ) are shown in the SI in Table S4. The individual seams are shown in Fig. 6. An intersection is considered bifurcating if *σ* < *Δ*_gh_ and in general when 
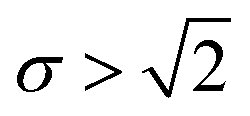
 then the intersection is sloped.^10^ And it is these sloped intersections that the literature consider can be avoided due to the descending nature of both paths.^10^ The other type of topology being peaked strongly influences the dynamics, where they attract and funnel the wavepacket down to the lower surface as the upper surface reaches a minima. The two other ways that the intersections can be categorised is whether they are bifurcating or single-path, the former resulting in multiple possible products on the lower surface and the latter results in a single product. One final point we will raise before the comparison is that this is all under an approximation first order linear model of the gradients and the nonadiabatic couplings of the two relevant states, so limitations can be expected in that context, but it has been shown to be satisfactory.^9,10,42,48^

**Fig. 6:**
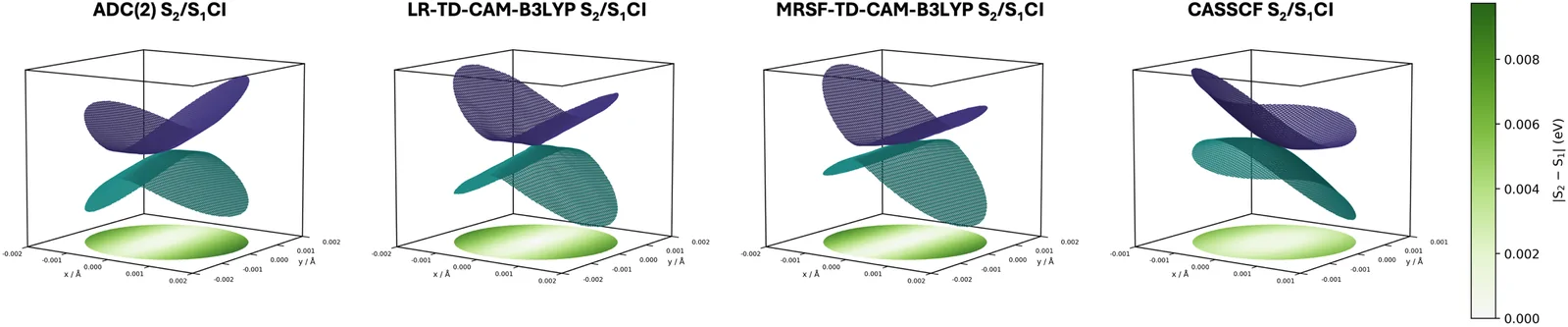
Topology of the conical intersections between the S_1_ and S_2_ states as calculated by, from left to right: ADC(2), LR-TD-CAM-B3LYP, MRSF-TD-CAM-B3LYP, and CASSCF.

Previously, we have pointed out the caveats of the single reference electronic structure methods for hopping to the ground state, this is not the case for the intersections between the excited states. From Fig. 6, the similarities between the S_2_ and S_1_ conical intersections can be observed. All the methods used for dynamics were categorised as a peaked bifurcating and all have a high degree of asymmetry (*Δ*_gh_ = 0.761–0.805), including CASSCF (which is categorised as sloped bifurcating).

In an attempt to determine why the MRSF-TD-CAM-B3LYP dynamics had more reported hops between S_1_ and S_2_ in terms of the intersection seam itself, we now compare the different parameters in Table S3. Given that the categorisation and grid framework is constructed within a first-order linear model within the 2D branching plane subspace, as *Δ*_gh_ → 1, there is the elongation along the *ŷ* direction, increasing the spatial range of the near-degeneracy. This in turn results in increased re-crossings, in an order that matches that of the observed average quantity of hops between S_1_ and S_2_ (as discussed in Section 3.3, from most to least: MRSF-TD-CAM-B3LYP (5.73 times, *Δ*_gh_ = 0.805), LR-TD-CAM-B3LYP (3.40 times, *Δ*_gh_ = 0.769), and ADC(2) (2.28 times, *Δ*_gh_ = 0.761). Additionally, MRSF-TD-CAM-B3LYP has the most shallow S_2_ minimum out of the different methods (largest 𝒫 (= 0.078) value). The increased time spent in the excited state could additionally rationalise the lower quantity of hops found to the ground state, as less time is spent where the nuclear configurational space allows for transition through the CI_1_.

#### 3.4.2 S_1_–S_0_ conical intersection topology

Let us now consider the actual topology of the ground and first excited states for both CI_1_ and CI_2_, to understand their role in a higher dimensional picture for their influence on dynamics, with the different levels of theory in Fig. 7. Here we include the methods used for dynamics (MRSF-TD-CAM-B3LYP, LR-TD-CAM-B3LYP, MP2/ADC(2)) as well as the reference method using CASSCF, note we do not include the MS-CASPT2 seam here for the reasons given in Section S10. Cyan represents the ground state and purple the first excited state.

**Fig. 7:**
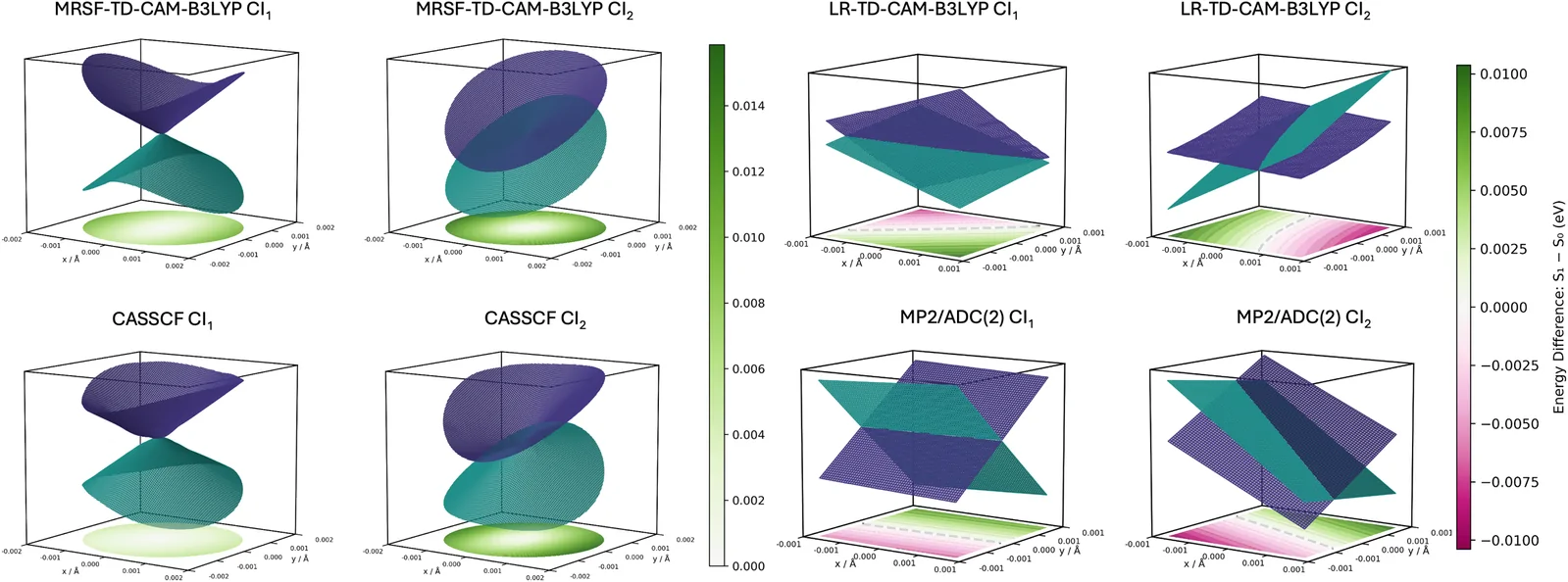
Topology of the different conical intersections CI_1_ and CI_2_ for various electronic structure methods, including CASSCF, MRSF-TDDFT, LR-TDDFT, and MP2/ADC(2). Cyan represents the ground state whilst purple indicates the first excited state.

Starting first with the single reference methods: LR-TDDFT and MP2/ADC(2). It is well known within the Hartree-Fock approximation that singly excited determinants do not mix directly with the ground state reference, as per Brillouin’s theorem,^50^ causing a breakdown in the definition in regions of degeneracy. Both LR-TDDFT and MP2/ADC(2) inherit analogous limitations. We find in the plotted seam that the branching space has a linear intersection of degeneracy, where past a point the first excited state (purple) becomes negative. Thus resulting in unphysical behaviour. Unlike in some previous studies,^15^ the LR-TDDFT seam is not as curved and more closely matches MP2/ADC(2). A reason to reinforce the criterion for preventing hops from S_1_ to S_0_ for LR-TDDFT, and perhaps ADC(2). Given the topology of the ground state for these levels of theory, we do not compute the 𝒫/ℬ values. This gives us a limited understanding on the influence of the conical intersection on the dynamics of the system, we can assume due to the geometric similarities between the conical intersections for these methods (see the RMSD in Table S7 the SI) and the multi-reference methods that their topologies will be more indicative of the influence on dynamics.

The diradical character of CI_1_ requires a description of an open-shell singlet ground state which becomes problematic for single reference methods. MRSF-TD-CAM-B3LYP inherently resolves this through the reference state, allowing the open-shell singlet ground state to be accessible through a simple spin-flip operation, as well as doubly-excited configurations. See Fig. S14 in the SI. We note that a recent study comparing LR-TDDFT and MRSF-TDDFT reported that the latter does not include certain singly excited configurations that may be relevant for some systems,^17^ yet improvements are being made such as the newly proposed Extended-MRSF method with additional configurations.^51^ As previously mentioned, MRSF-TDDFT is still a relatively new electronic-structure method, and further refinement and benchmarking will be important to fully understand its advantages and limitations for describing photochemical processes.

Given that, we now move on to the MRSF-TD-CAM-B3LYP and CASSCF CIs. For both versions of CI_1_ a peaked conical intersection is successfully described to great similarity. This was the CI present for all sets of dynamics, yet only correctly described by MRSF-TDDFT (out of the electronic structure methods used for dynamics). Inherently a peaked conical intersection has the classical double cone structure, where the saddle point of the intersection has a local minimum for the S_1_ state and a maximum for S_0_. Taking a look at the classification, see Table 2, indeed MRSF-TD-CAM-B3LYP (𝒫 = 0.012 and ℬ = 0.769) and CASSCF (𝒫 = 0.052 and ℬ = 0.827) are both categorised as peaked and bifurcating. This directly correlates with what is seen in dynamics, the bifurcating nature allowing for not just a single path to decay to on the ground state but in turn resulting in multiple photoproducts (those discussed in Section 3.2). The resulting photoproducts are dependent on many things when reaching the conical intersection, including the vibronic couplings and nuclear momenta.^48^

**Table 2:** Conical intersection topography parameters for CI_1_ and CI_2_. The pitch *δ*_gh_ (Hartree/Bohr), asymmetry *Δ*_gh_, relative tilt *σ*, and classification parameters 𝒫 and ℬ are defined following (ref. 10)

	CASSCF	MS-CASPT2	MRSF-TDDFT				
			CAM-B3LYP	BHHLYP	DTCAM-AEE	DTCAM-VEE	ωB97X-D
**CI_1_**							
*δ*_gh_	0.0460	0.0458	0.0519	0.0594	0.0497	0.0583	0.0692
*Δ*_gh_	0.288	0.955	0.154	0.216	0.172	0.246	0.713
*σ*	0.220	1.587	0.115	0.272	0.278	0.134	0.136
	0.052	2.982	0.012	0.072	0.072	0.015	0.014
	0.827	1.403	0.769	1.154	1.335	0.578	0.298
Classif.	P, B	S, SP	P, B	P, SP	P, SP	P, B	P, B

**CI_2_**							
*δ*_gh_	0.1205	0.7588	0.0722	0.0935	0.1164	0.1182	0.0577
*Δ*_gh_	0.443	0.994	0.066	0.330	0.590^a^	0.561^a^	0.620
*σ*	0.898	0.128	1.624	1.240	0.970	0.940	2.233
	1.444	2.867	2.802	2.291	1.203	1.302	6.766
	1.035	0.038	7.201	1.632	1.346^a^	1.353^a^	2.260
Classif.	S, SP	S, B	S, SP	S, SP	S, SP	S, SP	S, SP

a Near-symmetric intersection (*Δ*_gh_ ≈ 0); ℬ is unreliable in this limit.

CI_2_ for MRSF-TD-CAM-B3LYP and CASSCF both appears sloped with a definite main direction (single-path) for the wavepacket to travel on along the lower surface, this is confirmed with the classification in Table 2. The sloped nature of the intersection provides a useful insight into its role within dynamics. This intersection, presented as a hopping geometry within dynamics was found to be present only within ADC(2) dynamics. The sloped topology of CI_2_ rationalises its absence from both LR-TD-CAM-B3LYP and MRSF-TD-CAM-B3LYP dynamics, as sloped intersections are commonly bypassed.^9,10^ We can then attribute its presence to just ADC(2) dynamics to the artificially rising MP2 ground state,^52^ this results in a scenario in which there is no choice but for the intersection to be approached. This may seem like a hindrance to a researcher but could in turn be interpreted as a tool to find these sloped conical intersections that would otherwise be missed. Additionally from a non-quantitative point of view, the fast dynamics for ADC(2) provided a satisfactory geometric description of the conical intersections without having to propagate dynamics longer than 1 picosecond.

#### 3.4.3 XC functional dependence

We have found MRSF-TDDFT to be a useful alternative to single-reference methods such as LR-TDDFT and ADC(2), offering both reduced computational cost and a good description of the S_1_/S_0_ conical-intersection seam and the ground state. To our knowledge, the literature contains little direct comparison of how different XC functionals within MRSF-TDDFT perform in describing conical intersections. This system provides an excellent testing ground for such an analysis, given the two contrasting intersections with sloped and peaked topographies. Using the same procedure employed to generate the CASSCF and MRSF-TD-CAM-B3LYP intersections shown in Fig. 7, we obtain the results presented in Fig. 8.

**Fig. 8:**
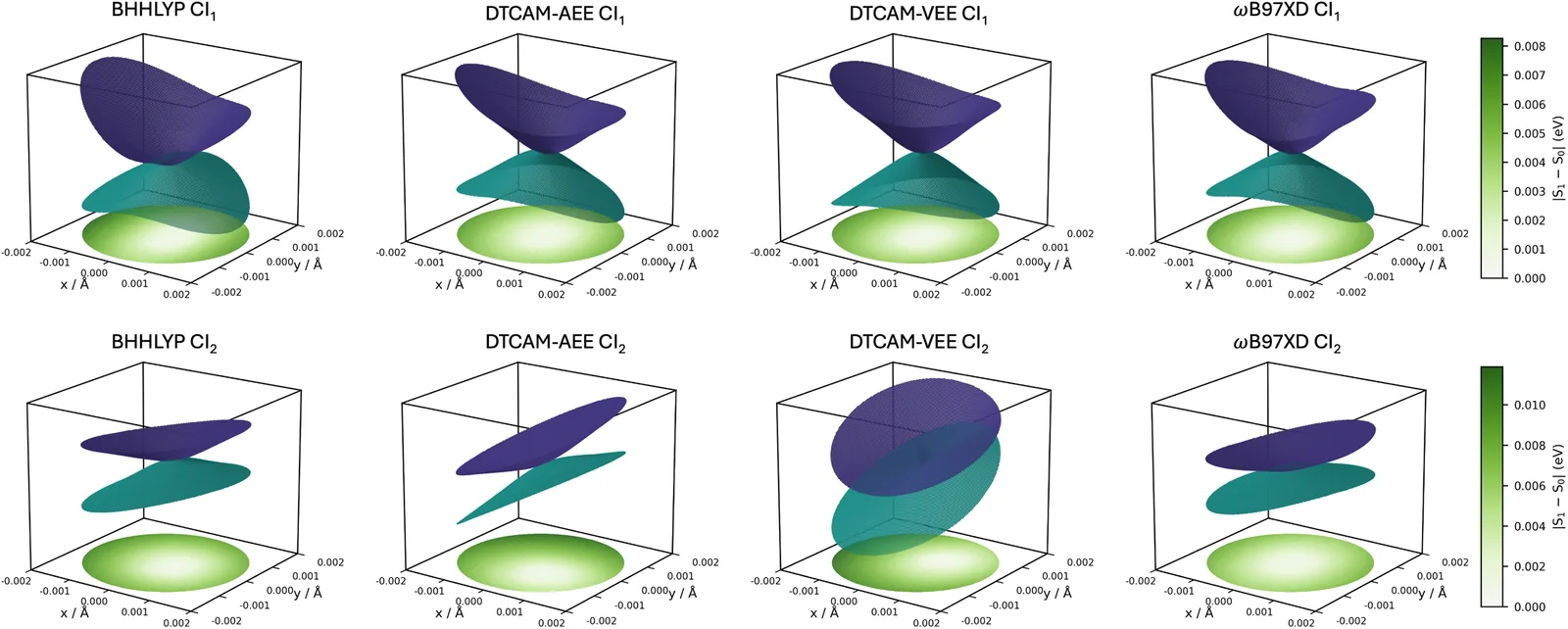
Topology of the different conical intersections CI_1_ and CI_2_ with MRSF-TDDFT for various XC functionals.

At least from visual inspection of Fig. 8, we can see the distinctions between CI_1_ and CI_2_ are consistent with that in the original plotted seam; CI_1_ retains the common peaked topology whilst the CI_2_ is consistently sloped. Since an inspection by eye limits us to an analysis of a static plot, we take advantage of the parameters 𝒫 and ℬ for the different XC functionals, including those calculated for CASSCF and CAM-B3LYP, in Table 2.

Consistently, CI_1_ is classified as a peaked conical intersection (𝒫 ≪ 1) for all methods except for MS-CASPT2, see Section S10 and Fig. S7, strongly correlating with the local minimum of the S_1_ potential energy surface around these nuclear coordinates. This is largely reinforced due to the small *σ* values (0.115 to 0.278). Additionally, the intersections for CI_1_ show more variation along the bifurcating/single-path axis. CAM-B3LYP (ℬ = 0.769), DTCAM-VEE (ℬ = 0.578), ωB97XD (ℬ = 0.298) and CASSCF (ℬ = 0.827) all give a bifurcated curvature (ℬ < 1), suggesting multiple relaxation pathways exist on the ground state, whereas BHHLYP (ℬ = 1.154) and DTCAM-AEE (ℬ = 1.335) give single-path. If ωB97XD, the most strongly bifurcating of the functionals, describes a genuinely bifurcating intersection, we would expect more than one outcome on the ground state rather than reformation of the starting geometry alone. We therefore performed dynamics on a subset of 20 trajectories using MRSF-TD-ωB97XD. When propagating up to two picoseconds, we found that the conical intersection (CI_1_) was indeed present in multiple trajectories. Whilst these trajectories experienced a similar problem to do with convergence issues as seen in MRSF-TD-CAM-B3LYP dynamics and previously described, there was still a reasonable quantity of ground state hops from the 20 trajectories (20%). Of the 20 trajectories 4 reached S_0_, all *via* CI_1_, with CI_2_ never accessed, consistent with the picture seen for MRSF-TD-CAM-B3LYP dynamics. The cleavage of the two symmetry-equivalent single bonds within the central cyclopropene unit was a near-even split for MRSF-TD-CAM-B3LYP trajectories. By contrast, for MRSF-TD-ωB97XD, 3 of the 4 trajectories reach S_0_*via* cleavage of the same bond, *r*_23_ within the first 700 fs; the remaining trajectory crosses to the ground state through the breaking of the other single bond, *r*_13_, at a considerably later time, *t* = 1553.5 fs. Examining the ground-state dynamics beyond the CI itself, 3 out of the 4 trajectories consequently show evidence of relaxing back toward the ring-closed, Franck–Condon like geometry. The remaining trajectory forms the diradical intermediate, as shown in the green box in Fig. 1; it does not form any of the photoproducts but maintains a level of closeness between the S_0_ and S_1_ energies suggesting it is stuck in a region of degeneracy, see Fig. S17. The persistence of the diradical, rather than immediate decay, is consistent with the bifurcating classification of CI_1_ at this functional, which permits more than one relaxation channel on the ground state. One thing not explored in this work is the influence of the varying active spaces and different flavours of CASSCF-based calculations as this has been shown to directly alter the outcome of the electronic structure results.^42,53^ This in-turn adds to the argument for more black-box based electronic structure methods, especially in benchmark cases.

CI_2_ on the other hand has agreement on all conical intersections being sloped with the requirement 𝒫 > 1, ranging from (1.203 to 6.766). As previously mentioned, if 
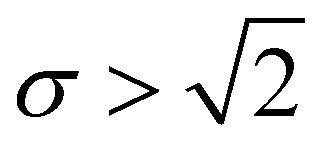
 is satisfied, which it is for CAM-B3LYP and ωB97XD, then regardless of other parameters it would be sloped.^10^ The sloped intersections seen here are commonly assumed to be bypassed and stay in the excited upper surface rather than undergoing a nonadiabatic transition.^9^ This notion reinforces the concept of its lack of a presence in the CAM-B3LYP MRSF-TDDFT based dynamics. The single-path classification is also consistent for all MRSF-TDDFT calculations (where ℬ > 1) indicating that if a nonadiabatic transfer did occur, there is only one preferred relaxation pathway.

The MS-CASPT2 result for CI_2_ also warrants a discussion, whilst the sloped classification is consistent with those from the MRSF-TDDFT functionals, MS-CASPT2 uniquely predicts the product to have a bifurcating character. This yields a sloped and bifurcating intersection, only possible when *Δ*_gh_ > ⅓.^10^ A recent study performed a similar analysis on varying DNA/RNA nucleobases, specifically using multiconfigurational wavefunction methods,^42^ a different flavour of CASSCF. Interestingly, only a minimal number of intersections calculated were classified as both sloped and bifurcating and when using the largest active space for each system, there was an absence of any sloped and bifurcating conical intersections. One could either conclude that a sloped and bifurcating conical intersection found with CAS based methods is rare or the active space used in this calculation is not enough. Given that active spaces of (12,12) and (10,10) were already reaching the computational limits for this system, we treat this conical intersection with caution; regardless, this is an interesting test case.

## 4 Conclusions

In this work, we have provided a large comparison of three key electronic structure methods used for the propagation of exploratory nonadiabatic dynamics to investigate the relevant conical intersections for this system. Ph_3_C_3_H undergoes nonradiative decay to the ground state through two different S_1_–S_0_ CIs with contrasting topologies, described only correctly through the use of multi-reference methods. CI_1_ is classified as peaked and bifurcating across CASSCF and most MRSF-TDDFT based XC functionals, with the exception of BHHLYP and DTCAM-AEE, which give single-path. CI_2_ on the other hand has a contrasting and consistent sloped-single-path topology for all MRSF-TDDFT functionals but appears sloped and bifurcating for MS-CASPT2. As expected, the single-reference methods cannot describe ground state correctly but excellently describe the S_2_/S_1_ conical intersections common to all dynamics.

The observed S_2_/S_1_ CI is peaked and bifurcating and facilitates a high level of contact between the two excited states throughout dynamics. We found the asymmetry parameter *Δ*_gh_ to correlate with the amount of hops between the two states. This is rationalised by the fact that this value approaching one results in the stretching of the area of degeneracy around the conical intersection, allowing for a higher chance of hopping between the surfaces. MRSF-TD-CAM-B3LYP experiences the least hops to the ground state, we largely attribute this to the combination of this CI trapping the population combined with the previously mentioned convergence issues.^17,32,33^ The subtle differences observed in the S_2_–S_1_ conical intersection topology with respect to the other levels of theory will be further explored in future work to determine whether they originate from the absence of certain excited configurations not included in the method.

In terms of the exploratory dynamics, all methods find CI_1_ as the primary decay pathway. MRSF-TD-CAM-B3LYP dynamics yields three outcomes (in order of frequency): initial geometry reformation, diradical intermediate, and the BC product. This indeed is facilitated through the bifurcating topology. Since MRSF-TD-CAM-B3LYP describes the ground state correctly, compared to the single reference method, it is recommended over LR-TDDFT for this class of system. This is with the caveat of the limitations of MRSF-TDDFT.^17^ If one wishes to perform exploratory dynamics, MP2/ADC(2) is a good alternative due to the fast nature of the dynamics and, although a negative of the method, finds the CI_2_ artificially due to the rising MP2 ground state. If combined with a higher level of theory, this can provide valuable insights into the system in question.

In addition to the comparison of the seams between the levels of theory, to our knowledge we also perform the first systematic analysis of XC functional dependence on the branching-plane topology parameters of S_1_/S_0_ conical intersections within MRSF-TDDFT. We find consistent agreement between functionals, for CI_1_ the small *σ* values reinforce this. Preliminary dynamics with ωB97XD yield both the reformed initial geometry and a persistent diradical intermediate, consistent with the bifurcating classification of CI_1_ at this functional. CI_2_ is consistently sloped for all XC functionals for MRSF-TDDFT. Across all of the S_1_/S_0_ CIs, the consistency for all MRSF-TDDFT XC functionals shows a robust use case for MRSF-TDDFT in describing the qualitative character of the seam in this class of systems. This provides a good black-box alternative to the complete active space methods that have proven to vary in results for excited state dynamics and conical intersection topologies when changing the variables of these calculations.^32,42,53^ The intramolecular nonradiative pathways and CI topologies identified here in the gas phase provide a foundation for future embedding calculations, to determine what changes happen when moving to the condensed phase in relation to the behaviour of Ph_3_C_3_H related rotors.

## Author contributions

M. Brady: data curation (lead); formal analysis (lead); investigation (equal); methodology (equal); software (lead); validation (lead); visualization (lead); writing – original draft (lead); writing – review and editing (equal). R. Crespo-Otero: conceptualization (lead); formal analysis (supporting); funding acquisition (lead); investigation (equal); methodology (equal); project administration (lead); resources (lead); supervision (lead); writing – original draft (supporting); writing – review and editing (lead).

## Conflicts of interest

There are no conflicts to declare.

## Supplementary Material

FD-OLF-D6FD00065G-s001

## Data Availability

Additional data, including descriptions of the calculations and tests performed at different levels of theory, details of the conical intersection analysis, and further information on the trajectories, are provided in the supplementary information (SI). The key geometry data supporting this article are available on Zenodo at https://doi.org/10.5281/zenodo.19632554. Supplementary information is available. See DOI: https://doi.org/10.1039/d6fd00065g.
