# Supplementary material for: Benchmarking electronic-structure methods for nonadiabatic dynamics in a propeller-shaped molecular rotor: insights into conical intersections
Source: Faraday Discuss. 2026 Oct 1. Online ahead of print. doi: 10.1039/d6fd00065g (PMC13629413; doi:10.1039/d6fd00065g)
Supplement: Supplementary file 1 [file FD-OLF-D6FD00065G-s001.pdf]

Supplementary Information (SI) for Faraday Discussions.
This journal is © The Royal Society of Chemistry

Supplementary Information

Benchmarking electronic-structure methods for nonadiabatic dynamics in a propeller-shaped molecular rotor: Insights into conical intersections

Marcus Brady,[a] Rachel Crespo-Otero[a*]

[a] Department of Chemistry, University College London, 20 Gordon Street, WC1H 0AJ London, United Kingdom
*Corresponding author: r.crespo-otero@ucl.ac.uk

S1 CASSCF Orbitals

S1.1 (12,12)

Figure S1 shows the 12 orbitals used for the 6 state averaged CASSCF calculations. With the def2-SVP basis set. These orbitals were used for all CAS calculations. The [10,10] active space used in the MS-CASPT2 calculations were symmetric around the HOMO/LUMO (i.e. 67 to 76).

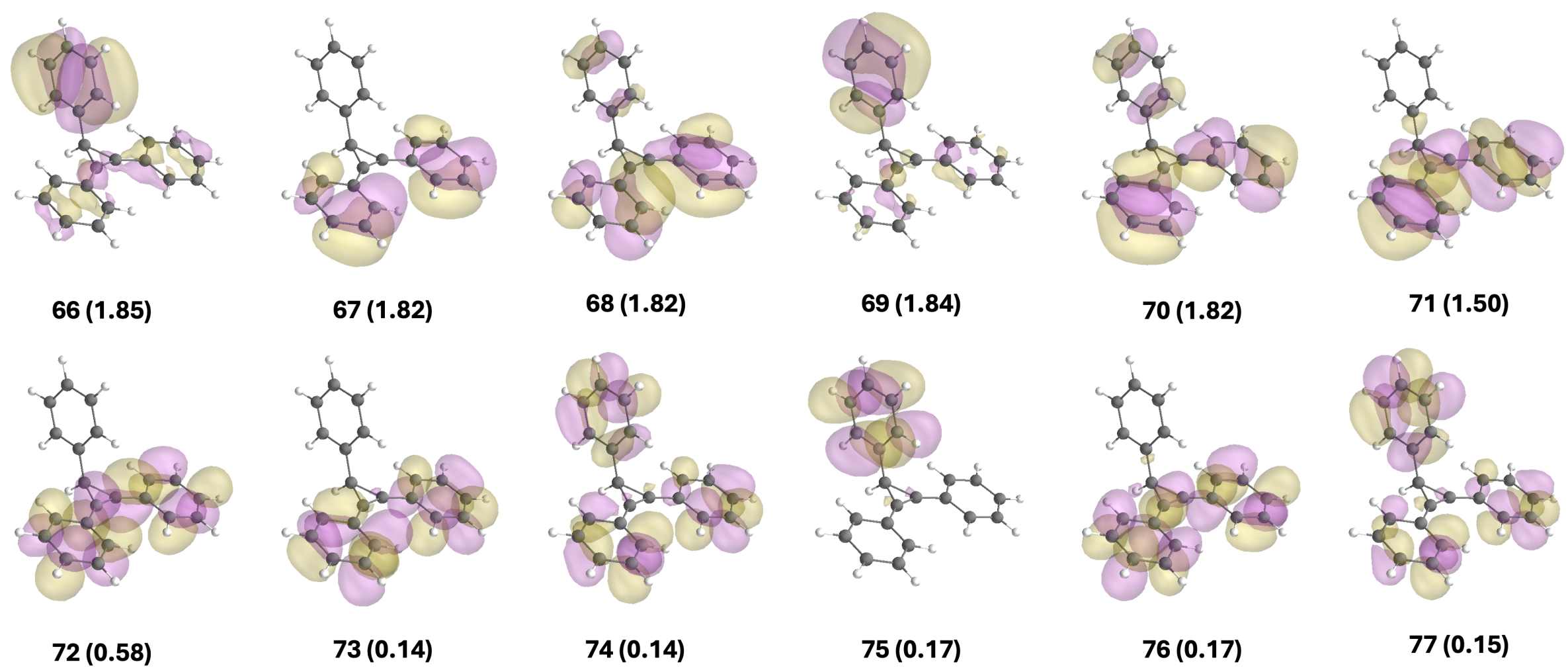

Figure S1: The 12 orbitals and their respective occupation.

S2 Basis Set Convergence Test

Table S1 shows the vertical excitations obtained with different basis sets.

Table S1: MRSF-TD-CAM-B3LYP calculations with different basis sets for the first three excited states (in eV). The oscillator strengths are included in brackets.

	cc-pVDZ	cc-pVTZ	def2-SVP	def2-TZVP	6-31G*	6-31G**
S_1 ($\pi\pi^*$)	4.13 (1.31)	4.09 (1.30)	4.14 (1.31)	4.08 (1.30)	4.18 (1.32)	4.18 (1.32)
S_2 ($\pi\pi^*$)	4.38 (0.03)	4.34 (0.03)	4.38 (0.03)	4.34 (0.03)	4.40 (0.03)	4.40 (0.03)
S_3 ($\pi\pi^*$)	5.12 (0.00)	5.02 (0.01)	5.12 (0.00)	4.99 (0.01)	5.18 (0.01)	5.18 (0.00)

S3 CASSCF and MS-CASPT2 Vertical Excitations

Table S2: Vertical excitation energies (eV) the S_0 minimum energy geometry for the first three excited states. The basis set for all calculations was def2-SVP.

	Exp.	SA(3)-CASSCF	MS(3)-CASPT2
**FC Geom**		**(12,12)**	**(10,10)**
S_1 ($\pi\pi^*$)	3.75	4.69	4.86
S_2 ($\pi\pi^*$)	3.95	5.59	4.86
S_3 ($\pi\pi^*$)	–	6.09	–

S4 Generation of grid around the conical intersection

Due to the complexity of some of the conical intersections and their seams, different grid ranges and resolutions were employed to achieve the most accurate representation of the conical intersection seams. For the S_2/S_1 conical intersections (shown in Figure 6 in the main text), ADC(2), LR-TD-CAM-B3LYP, and MRSF-TD-CAM-B3LYP calculations used grids spanning ± 0.002 with step sizes of 0.0004. In contrast, the CASSCF grid was slightly narrower, covering ± 0.001 with the same step size of 0.0004. For the key CI_1 and CI_2 intersections computed with MRSF-TD-CAM-B3LYP, a finer grid of ± 0.002 with step sizes of 0.0002 was used, corresponding to a resolution of 21×21 points. A similar 21×21 grid was employed for the single-reference methods; however, a reduced range of ± 0.001 with step sizes of 0.0001 was used due to instabilities in the ground-state calculations. The CASSCF intersections were evaluated at a lower resolution, using a grid of ± 0.002 with step sizes of 0.0005, reflecting the higher computational cost of these calculations. Finally, the different exchange–correlation functionals within MRSF-TDDFT were all computed using grids spanning ± 0.002 with step sizes of 0.0004.

S5 Generation of the conical intersection grid

S5.1 Grid Calculation

We first start with the two branching vectors $\mathbf{g}_{ij}$ (gradient difference vector between states i and j) and $\mathbf{h}_{ij}$ (nonadiabatic coupling vector between states i and j). These vectors undergo Yarkony rotation through β to make them orthogonal:

$$\bar{\mathbf{g}}_{ij} = \mathbf{g}_{ij} \cos(2\beta) + \mathbf{h}_{ij} \sin(2\beta), \tag{S1}$$

$$\bar{\mathbf{h}}_{ij} = \mathbf{h}_{ij}\cos(2\beta) + \mathbf{g}_{ij}\sin(2\beta). \tag{S2}$$

Where these "barred" variations of the branching vectors make sure the seam is continuous along the seam by construction.[1] The solution for β is trivial under the requirement $\bar{\mathbf{h}}_{ij} \cdot \bar{\mathbf{g}}_{ij} = 0$, as outlined in Ref[1, 2]:

$$\frac{\|\mathbf{h}_{ij}\|^2 - \|\mathbf{g}_{ij}\|^2}{2}\sin 4\beta - [\mathbf{h}_{ij} \cdot \mathbf{g}_{ij}]\cos 4\beta = \bar{\mathbf{h}}_{ij} \cdot \bar{\mathbf{g}}_{ij} = 0 \tag{S3}$$

and for when $||\mathbf{h}_{ij}\| \neq |\mathbf{g}_{ij}\|$,

$$\tan 4\beta = \frac{2[\mathbf{h}_{ij} \cdot \mathbf{g}_{ij}]}{[\|\mathbf{h}_{ij}\|^2 - \|\mathbf{g}_{ij}\|^2]}, \tag{S4}$$

for the solutions $\beta = \beta_0 + (m\pi)/4$ for $m \in \mathbb{Z}^*$. The normalised branching vectors are referred to as $\bar{\mathbf{x}}_{ij}$ and $\bar{\mathbf{y}}_{ij}$ for $\bar{\mathbf{g}}_{ij}$ and $\bar{\mathbf{h}}_{ij}$ respectively. Once obtained, a grid was generated ($\mathbf{R}(x, y)$) around the conical intersection ($\mathbf{R}$), perturbed by a small arbitrary length (x and y) along the branching space vectors.

$$\mathbf{R}(x, y) = \mathbf{R} + x\bar{\mathbf{x}}_{ij} + y\bar{\mathbf{y}}_{ij}. \tag{S5}$$

S5.2 Parameter Calculation

In addition to constructing branching-plane grids to map the conical intersection seams, the branching vectors, along with several related quantities, can be used to establish a classification scheme for different types of conical intersections. Previous studies have provided clear frameworks for this type of classification,[3, 4] and, for completeness, we briefly summarise the approach here. We begin by considering the orthogonalised gradient difference vector ($\bar{\mathbf{g}}_{ij}$) and the nonadiabatic coupling vector ($\bar{\mathbf{h}}_{ij}$), together with the gradient average, defined as:

$$\mathbf{s}^{AB} = \frac{1}{2}\left(\nabla E_B + \nabla E_A\right) \tag{S6}$$

$$\delta_{gh} = \sqrt{\frac{1}{2}\left(\|\bar{\mathbf{g}}\|^2 + \|\bar{\mathbf{h}}\|^2\right)} \tag{S7}$$

$$\Delta_{gh} = \frac{\|\bar{\mathbf{g}}\|^2 - \|\bar{\mathbf{h}}\|^2}{\|\bar{\mathbf{g}}\|^2 + \|\bar{\mathbf{h}}\|^2} \qquad \Delta_{gh} \in [0, 1] \tag{S8}$$

Calculating the branching vectors ($\hat{x}$ and $\hat{y}$) as outlined in the previous section.

$$s_x = \frac{\mathbf{s}^{AB} \cdot \hat{x}}{\delta_{gh}} \qquad s_y = \frac{\mathbf{s}^{AB} \cdot \hat{y}}{\delta_{gh}} \tag{S9}$$

$$\sigma = \sqrt{s_x^2 + s_y^2} \qquad \tan(\theta_s) = \frac{s_y}{s_x} \tag{S10}$$

We now give a quick overview of the calculation of the different parameters. Using the branching vectors obtained as previously described, parameters can be obtained to classify the topologies of the conical intersections in terms of the peaked/sloped and bifurcated/single-path nature, denoted as $\mathcal{P}$ and $\mathcal{B}$ and are calculated as:

$$\mathcal{P} = \frac{\sigma^2}{1-\Delta_{gh}^2}\left(1-\Delta_{gh}\cos 2\theta_s\right)\begin{cases} <1 & \text{Peaked} \\ >1 & \text{Sloped}\end{cases} \tag{S11}$$

$$\mathcal{B} = \sqrt[3]{\frac{\sigma^2}{4\Delta_{gh}^2}}\left(\sqrt[3]{(1+\Delta_{gh})\cos^2\theta_s}+\sqrt[3]{(1-\Delta_{gh})\sin^2\theta_s}\right)\begin{cases} <1 & \text{Bifurcating} \\ >1 & \text{Single-Path}\end{cases} \tag{S12}$$

There are some essential conditions that should be noted as outlined in Ref.[3] Sloped and bifurcating intersections are only possible if $\Delta_{gh} > 1/3$; below this, the bifurcating character is mathematically impossible and the parameter $\mathcal{B}$ is unreliable.

S6 Local Diabatisation

S6.1 Computational Details

The Newton-X implementation of wavefunction overlap was used to compute the nonadiabatic couplings between electronic states within LR-TDDFT.[5] This analysis was performed on five randomly sampled trajectories generated from the LR-TD-CAM-B3LYP initial conditions. The aim was purely exploratory, namely to assess whether the observed transitions from S_1 to S_2 and higher states could be artefacts of the TD-BA approximation.[6]

S6.2 Subsampled Trajectories

Out of the 5 trajectories, 3 showed hops between the S_1 and S_2 and we include an example trajectory below in terms of the energy and the hops over time below. In fact, the local-diabatised surface hops sooner than the TD-BA.

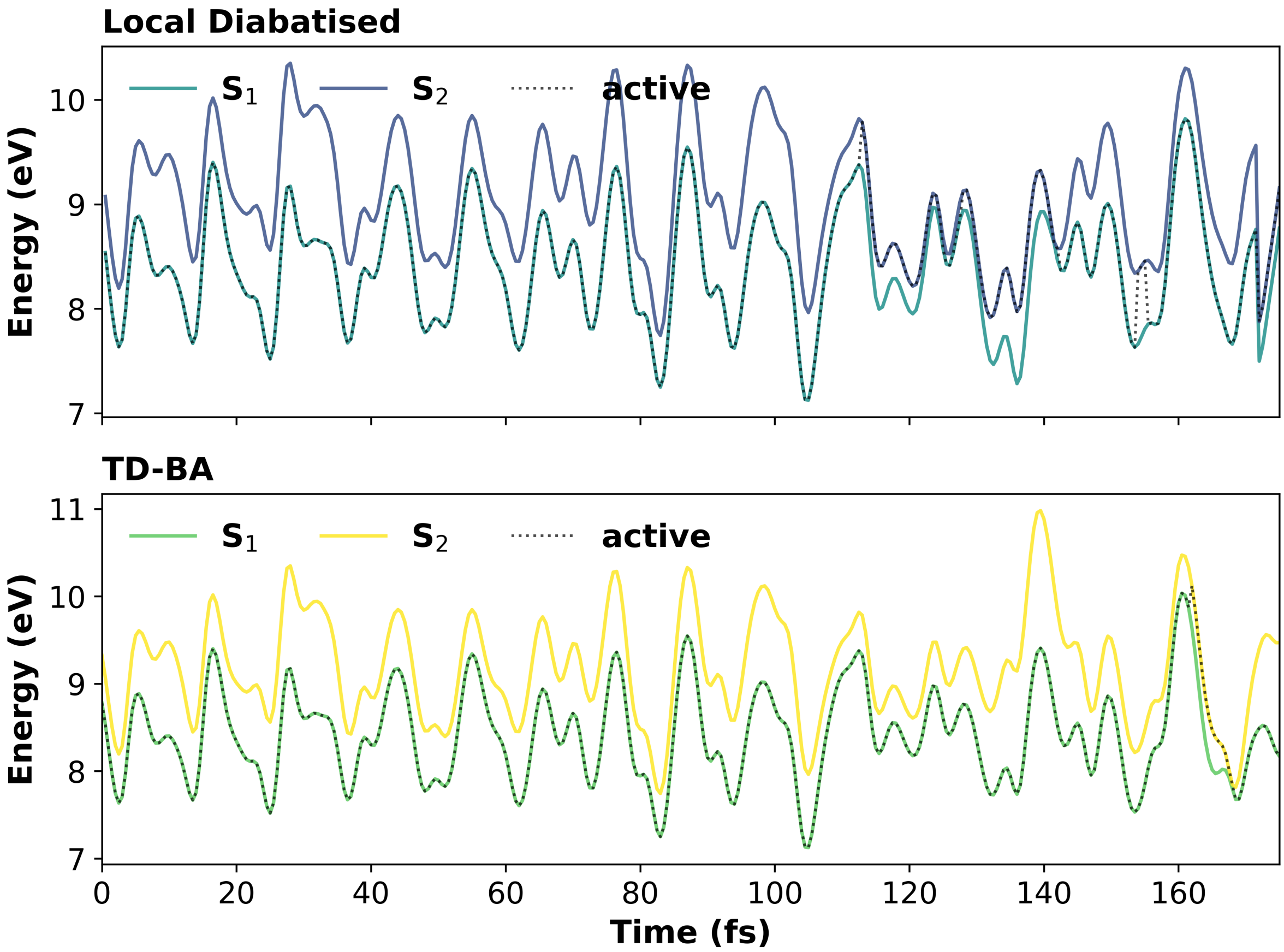

Figure S2: Example trajectory with many hops between S_1 and S_2 for the wavefunction overlapped dynamics for LR-TD-CAM-B3LYP/def2-SVP compared to the TD-BA.

S7 Absorption Spectra

Figure S3 shows the absorption spectrum generated from the initial conditions using the nuclear ensemble approach. The relevant electronic structure methods employed in the dynamics are indicated. In this spectrum, we include the combined contribution of the S_1, S_2, and S_3 manifolds as an overall shifted spectrum. We also present the spectrum decomposed into the individual state contributions in Figure S4.

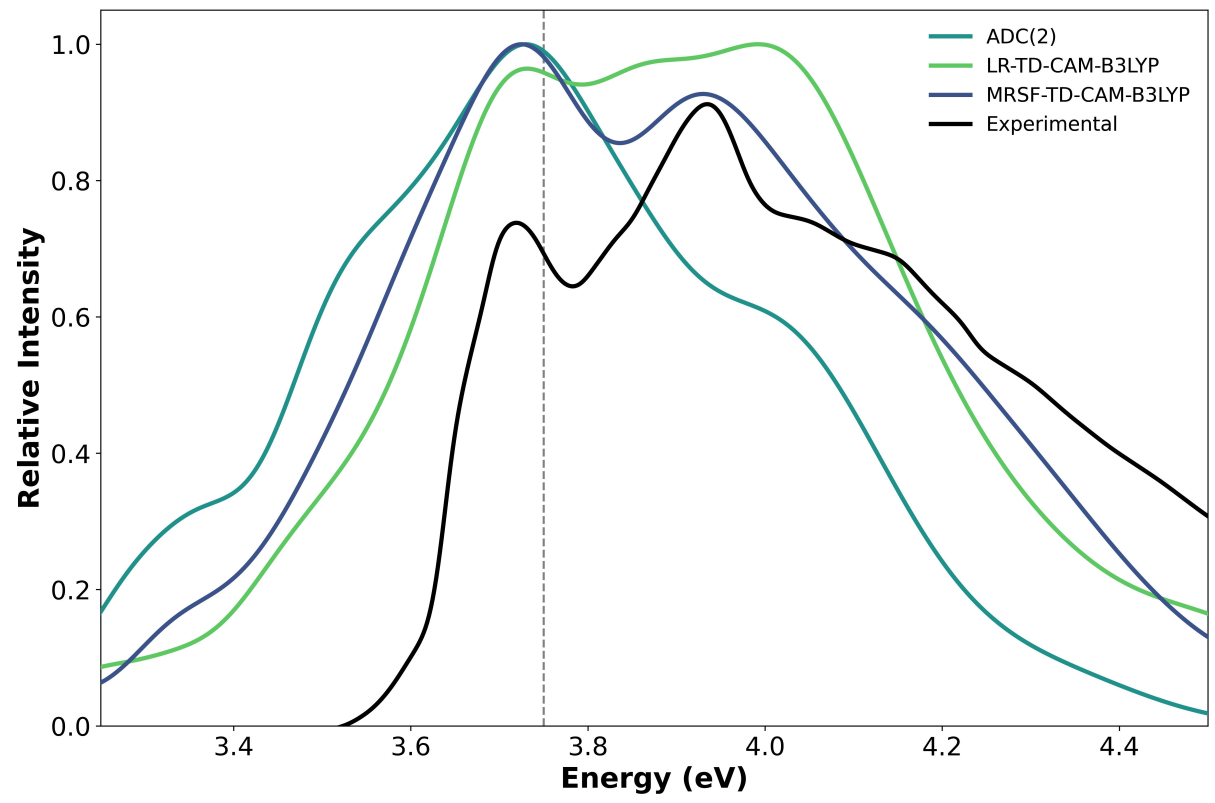

Figure S3: Absorption spectra with the relevant levels of theory with the experimental data.[7]

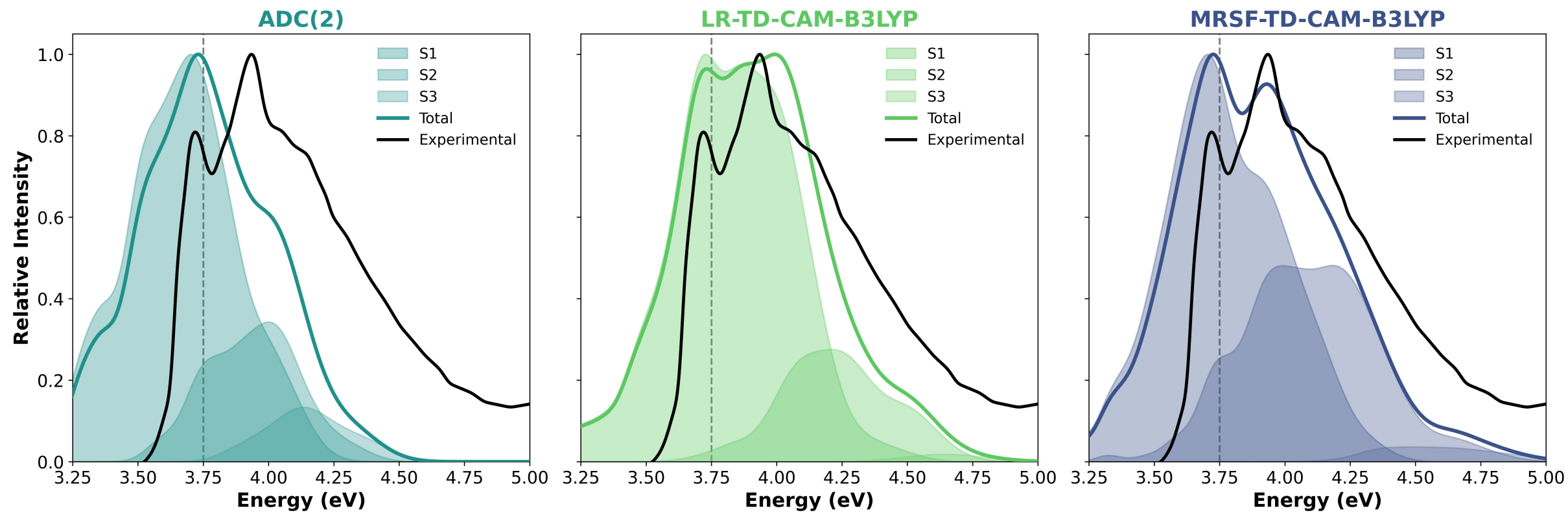

Figure S4: Absorption spectra with the relevant levels of theory with the experimental data, including the individual excited state contributions.[7]

S8 Key Geometries

The key bond lengths of the S_0 and S_1 minimum-energy geometries were obtained from (TD-)DFT optimisations at the CAM-B3LYP/def2-SVP level. The structure optimised at the CASSCF level (using the previously described active space) preserves the single C–C bond length at 1.50 Å, while the double bond shortens from 1.31 Å to 1.28 Å.

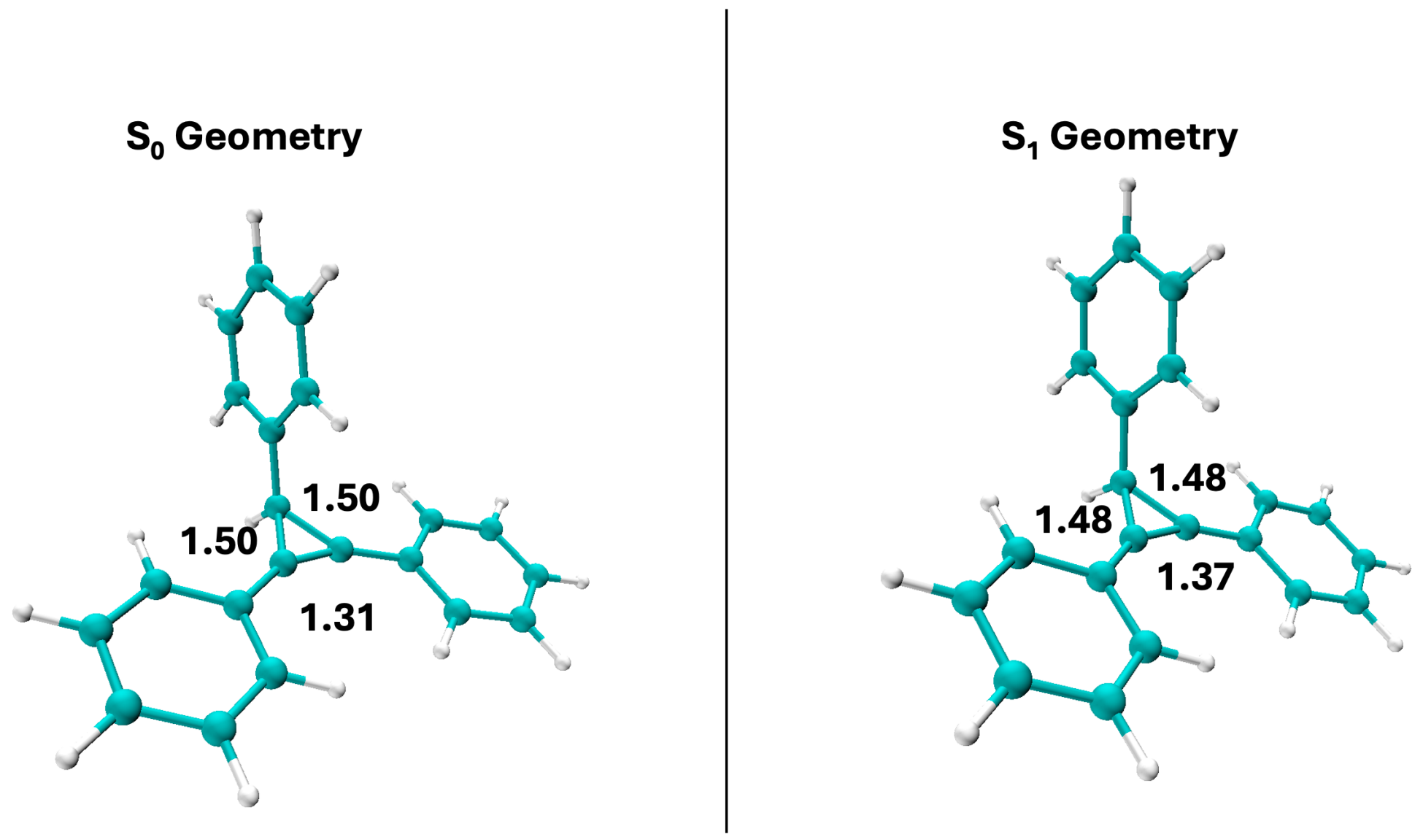

Figure S5: S_0 and S_1 minimum energy geometries as optimised with (TD-)DFT

S9 The S_2S_1 CI

S9.1 The geometries

Figure S6 shows the optimised S_2/S_1 conical intersection, which is not far from the Franck–Condon geometry.

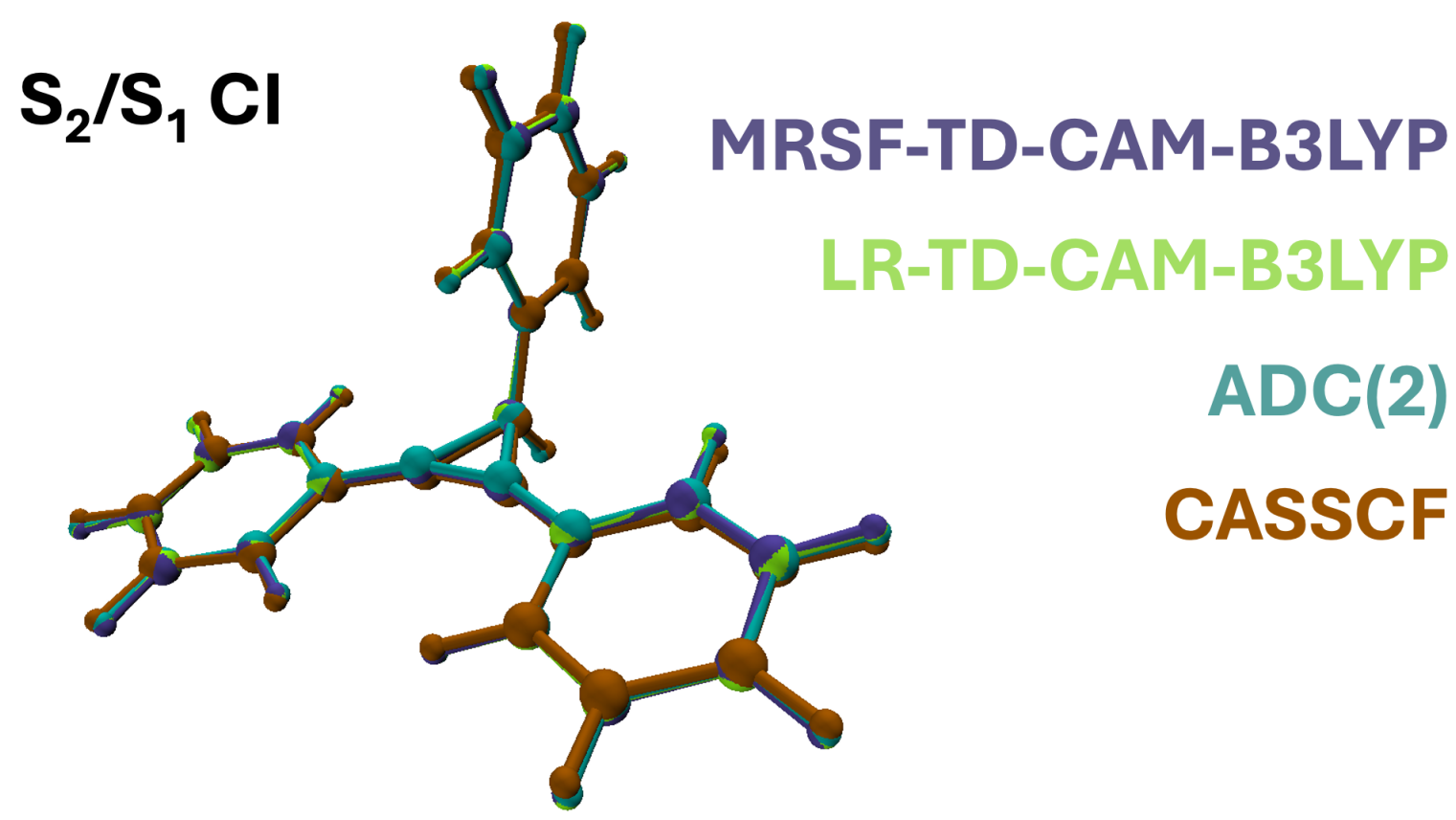

Figure S6: The optimised S_2/S_1 for the considered electronic structure methods.

RMSD Parameters

Table S3: RMSD (Å) of each method relative to MRSF-TD-CAM-B3LYP, CASSCF, and PT2 at the S_2/S_1 CI.

	**MRSF-TD-CAM-B3LYP**	**CASSCF**	**MS-CASPT2**
**ADC(2)**	0.025	0.094	0.061
**CASSCF**	0.092	0.000	0.061
**LR-TD-CAM-B3LYP**	0.003	0.091	0.058
**MRSF-TD-CAM-B3LYP**	0.000	0.092	0.059
**MS-CASPT2**	0.059	0.063	0.000

S9.2 Parameters

For Table S4, we report the relevant parameters used to classify the conical intersection topology. All cases, except CASSCF, are classified as peaked and bifurcating.

Table S4: Conical intersection topography parameters for the S_2/S_1 CI. We include the pitch δ_{gh} (Hartree/Bohr), asymmetry Δ_{gh}, relative tilt σ, and classification parameters $\mathcal{P}$ and $\mathcal{B}$.

	**CASSCF**	**MRSF-TDDFT**	**LR-TDDFT**	**ADC(2)**
δ_{gh}	0.0973	0.0650	0.0611	0.0616
Δ_{gh}	0.633	0.805	0.769	0.761
σ	0.748	0.366	0.184	0.231
$\mathcal{P}$	1.367	0.078	0.019	0.030
$\mathcal{B}$	0.903	0.493	0.307	0.359
Classif.	S, B	P, B	P, B	P, B

S10 Geometric Parameters for the S_1/S_0 CI_1/CI_2 structures

In Tables S5 and S6, we include the key reaction coordinates outlined in the main text, tabulated for each level of theory.

Table S5: Bond lengths (Å) at CI_1 and CI_2 for each method.

	**C1-C2**		**C1-C3**		**C2-C3**	
	CI_1	CI_2	CI_1	CI_2	CI_1	CI_2
**ADC(2)**	1.326	1.545	1.448	1.476	2.352	1.762
**LR-TD-CAM-B3LYP**	1.373	1.550	1.396	1.424	2.354	1.665
**MRSF-TD-CAM-B3LYP**	1.372	1.549	1.397	1.430	2.353	1.658
**CASSCF**	1.441	1.466	1.350	1.413	2.472	1.830
**MS-CASPT2**	1.360	1.493	1.436	1.462	2.474	1.871

Table S6: Dihedral angles (degrees) at CI1 and CI2 for each method.

	$\boldsymbol{\phi_1}$		$\boldsymbol{\phi_2}$		$\boldsymbol{\phi_3}$	
	CI_1	CI_2	CI_1	CI_2	CI_1	CI_2
**ADC(2)**	−67.5	−158.2	113.7	149.3	−21.0	71.2
**LR-TD-CAM-B3LYP**	−74.7	−152.1	109.6	146.9	−25.9	78.1
**MRSF-TD-CAM-B3LYP**	−74.7	−152.3	109.4	146.9	−26.0	78.5
**CASSCF**	−60.0	−150.0	115.6	134.7	−29.0	61.7
**MS-CASPT2**	−40.8	−150.0	129.5	134.7	−30.5	61.7

RMSD Parameters for CI_1 and CI_2

Table S7: RMSD (Å) of each method relative to MRSF-TD-CAM-B3LYP, CASSCF, and PT2 at CI_1 and CI_2.

	MRSF-TD-CAM-B3LYP		CASSCF		MS-CASPT2	
	CI_1	CI_2	CI_1	CI_2	CI_1	CI_2
**ADC(2)**	0.046	0.044	0.309	0.376	0.437	0.413
**CASSCF**	0.310	0.378	0.000	0.000	0.211	0.143
**LR-TD-CAM-B3LYP**	0.000	0.002	0.310	0.378	0.444	0.415
**MRSF-TD-CAM-B3LYP**	0.000	0.000	0.310	0.378	0.444	0.415
**MS-CASPT2**	0.444	0.415	0.211	0.143	0.000	0.000

Brief description on multireference optimisations and MS-CASPT2 CIs

The MS-CASPT2-based S_1/S_0 conical intersection optimisations performed with three-state averaging in this study did locate intersections, with degeneracies of 0.0363 eV and 0.0192 eV. However, the corresponding seams proved difficult to characterise. It has previously been noted that variations in CASSCF, based computational parameters, such as the choice of active space, orbital occupations, and state averaging, can significantly affect both the classification and the topology of conical intersection seams.[8, 9] Furthermore, different flavours of CASSCF can directly influence the outcomes of nonadiabatic dynamics.[10] A systematic, static investigation of active spaces, occupations, and state-averaging schemes would therefore be required for a comprehensive comparison of the resulting topologies.

Figure S7 shows the two distinct seams obtained with three-state-averaged MS-CASPT2[10,10]. As highlighted in the main text, these differ from the other methods, particularly in the case of CI_1. We anticipate that exploring alternative active spaces would likely lead to different results, as discussed previously.[8]

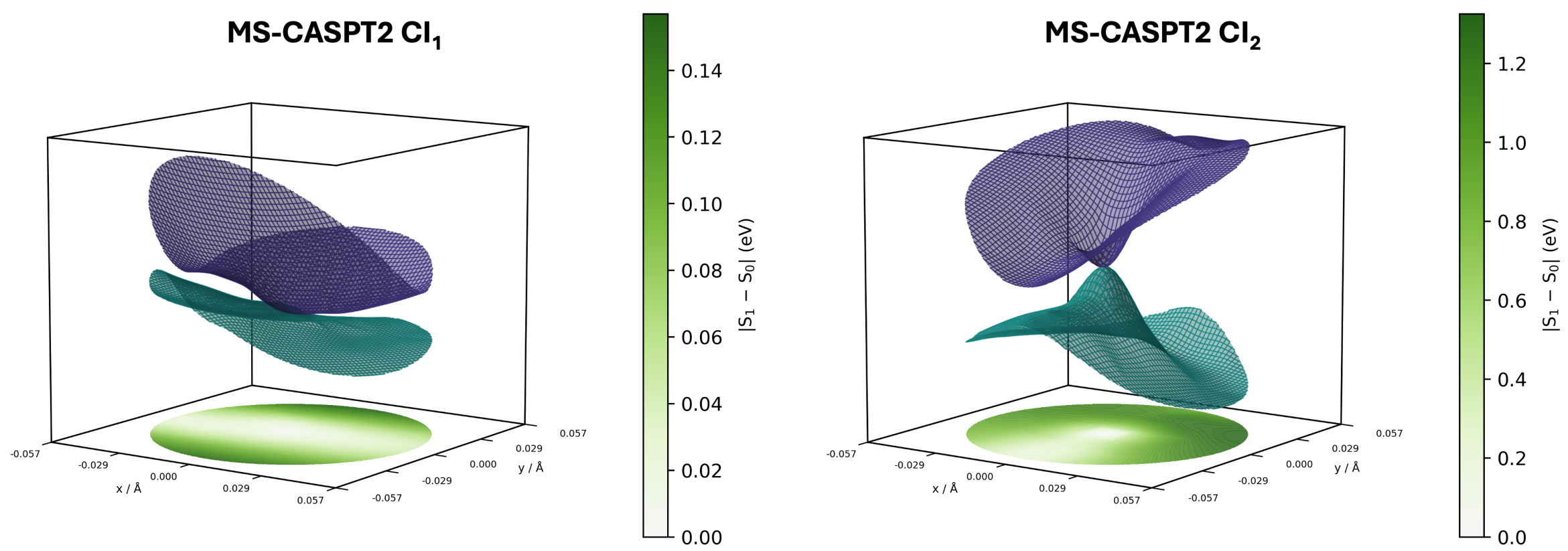

Figure S7: The MS-CASPT2 seams for CI_1 and CI_2, a 6 by 6 grid from ±0.05 Å with step sizes of 0.02Å. We additionally include the origin point due to the sparsity of grid due to computational cost of the large active space with the quantity of atoms.

S11 Photoproduct Example Data

In Figure S8, we present an example trajectory from MP2/ADC(2) dynamics, illustrating a pathway involving the S_1/S_0 CI_2 that is unique to ADC(2). Through the torsion of the two phenyl rings located to the left and right of the central cyclopropene ring, the MP2 ground-state energy increases, thereby enabling access to this crossing. Notably, the three central carbon atoms associated with ring opening show little to no fluctuation along this pathway.

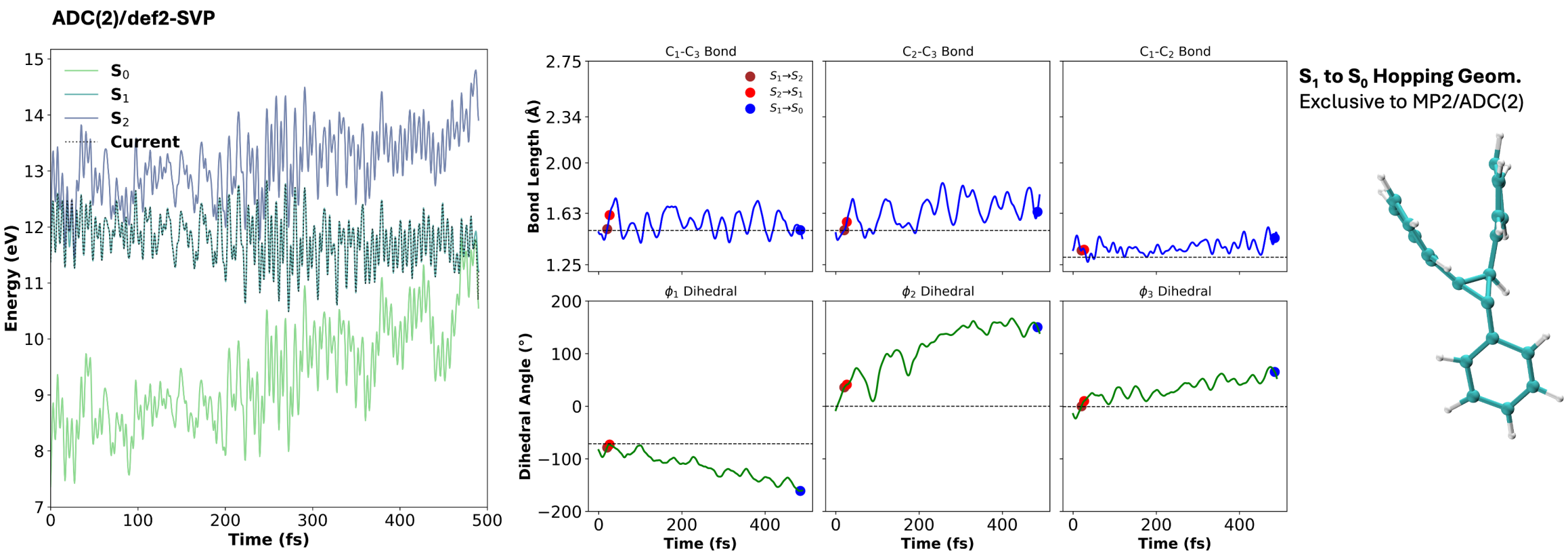

Figure S8: Example trajectory to show the evolution of energy and reaction coordinates over time for an MP2/ADC(2) trajectory.

S12 Reaction Coordinates

To analyse the mechanistic details of the different sets of dynamics, we plot the average values of the key reaction coordinates up to 1500 fs in Figure 4 of the main text. When fewer than half of the trajectories contribute to the average, the corresponding lines are shown as transparent. The dotted horizontal line indicates the equilibrium value of each reaction coordinate.

The figures below show the individual key reaction coordinates for all trajectories at each level of theory. For the single-reference methods, we indicate ground-state hopping events, while for MRSF-TD-CAM-B3LYP we include $S_0 \rightarrow S_1$ transitions.

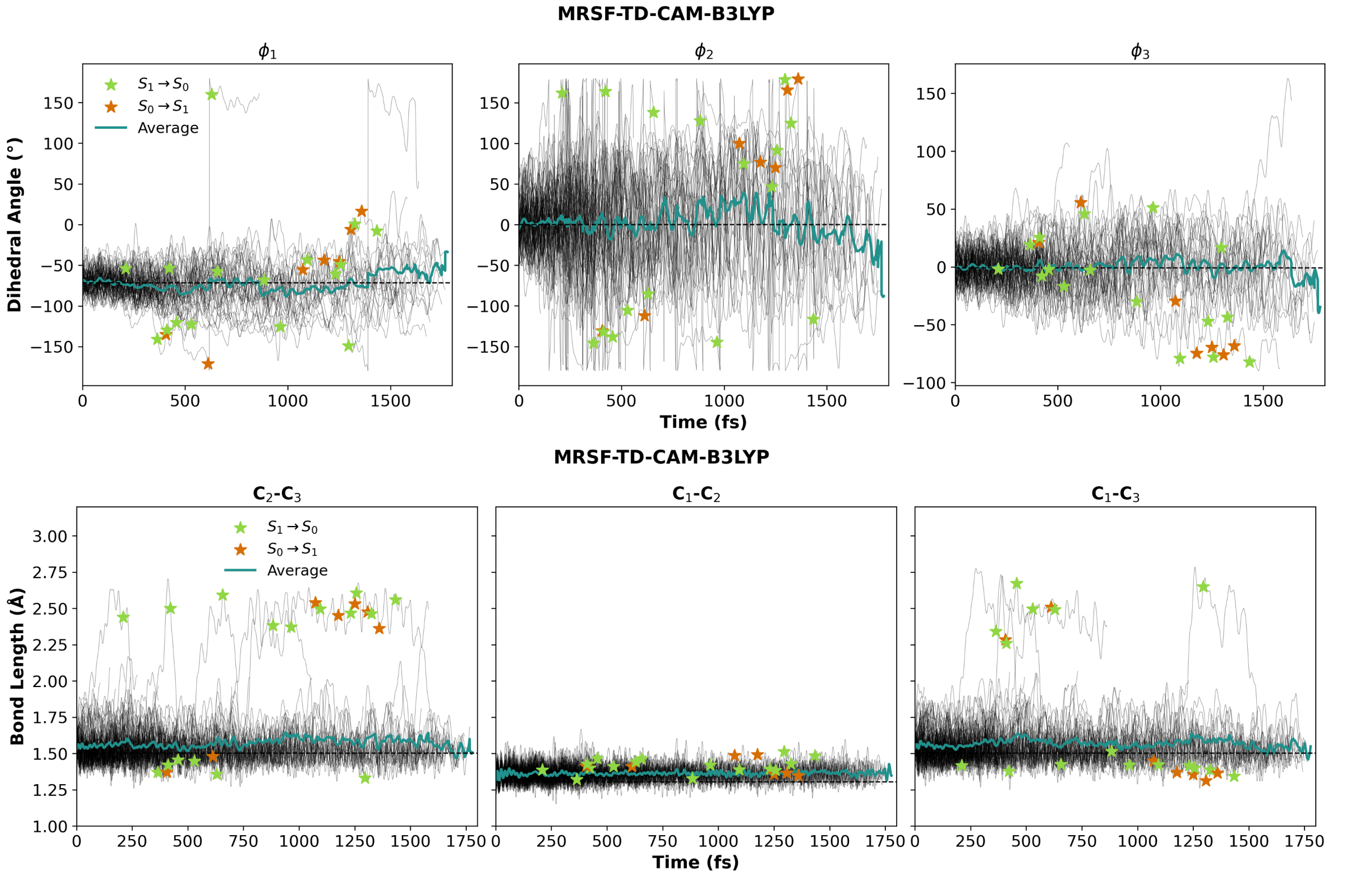

Figure S9: Reaction coordinates for all trajectories, with the respective hops for MRSF-TD-CAM-B3LYP based dynamics

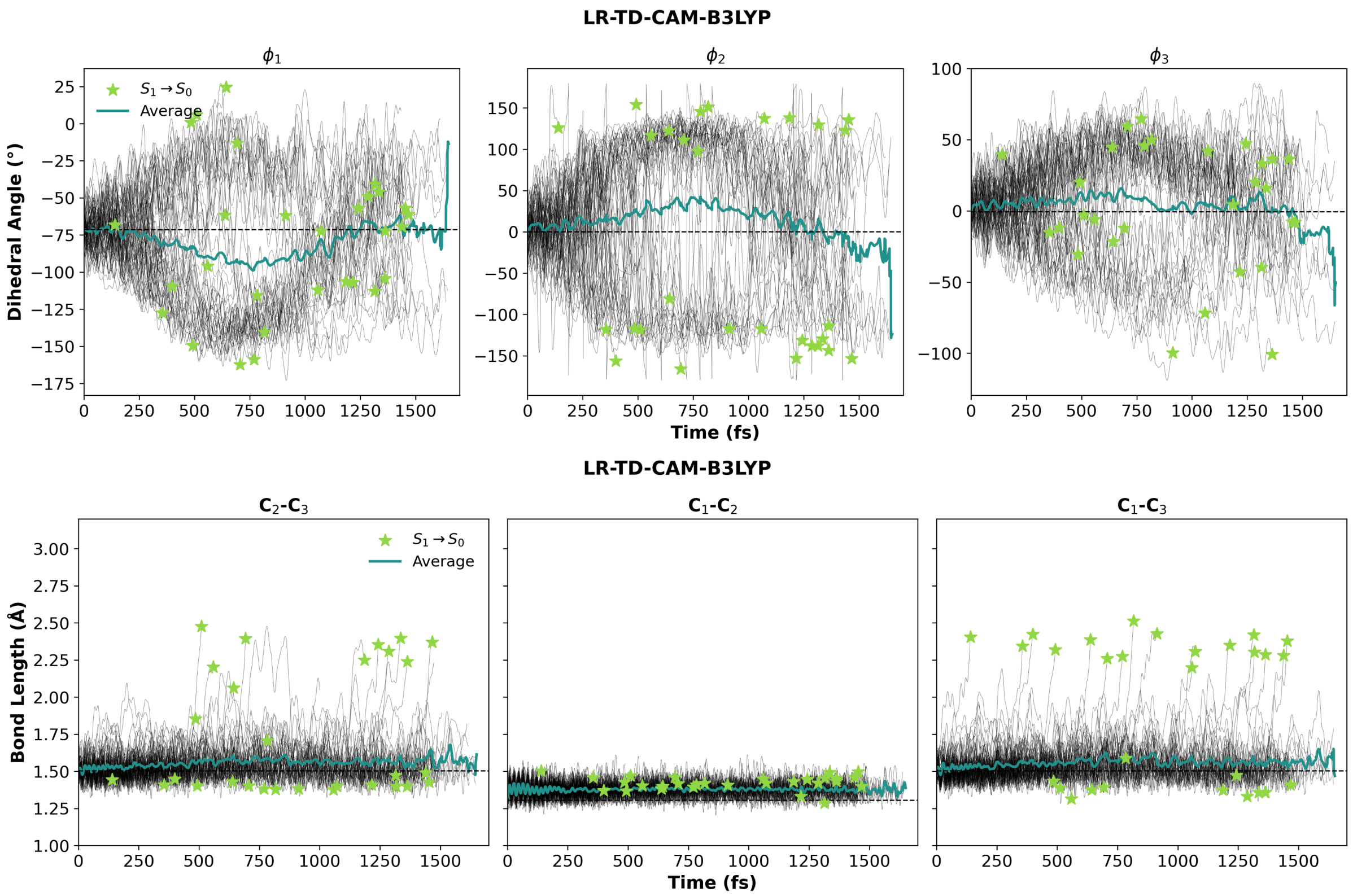

Figure S10: Reaction coordinates for all trajectories, with the respective hops for LR-TD-CAM-B3LYP based dynamics

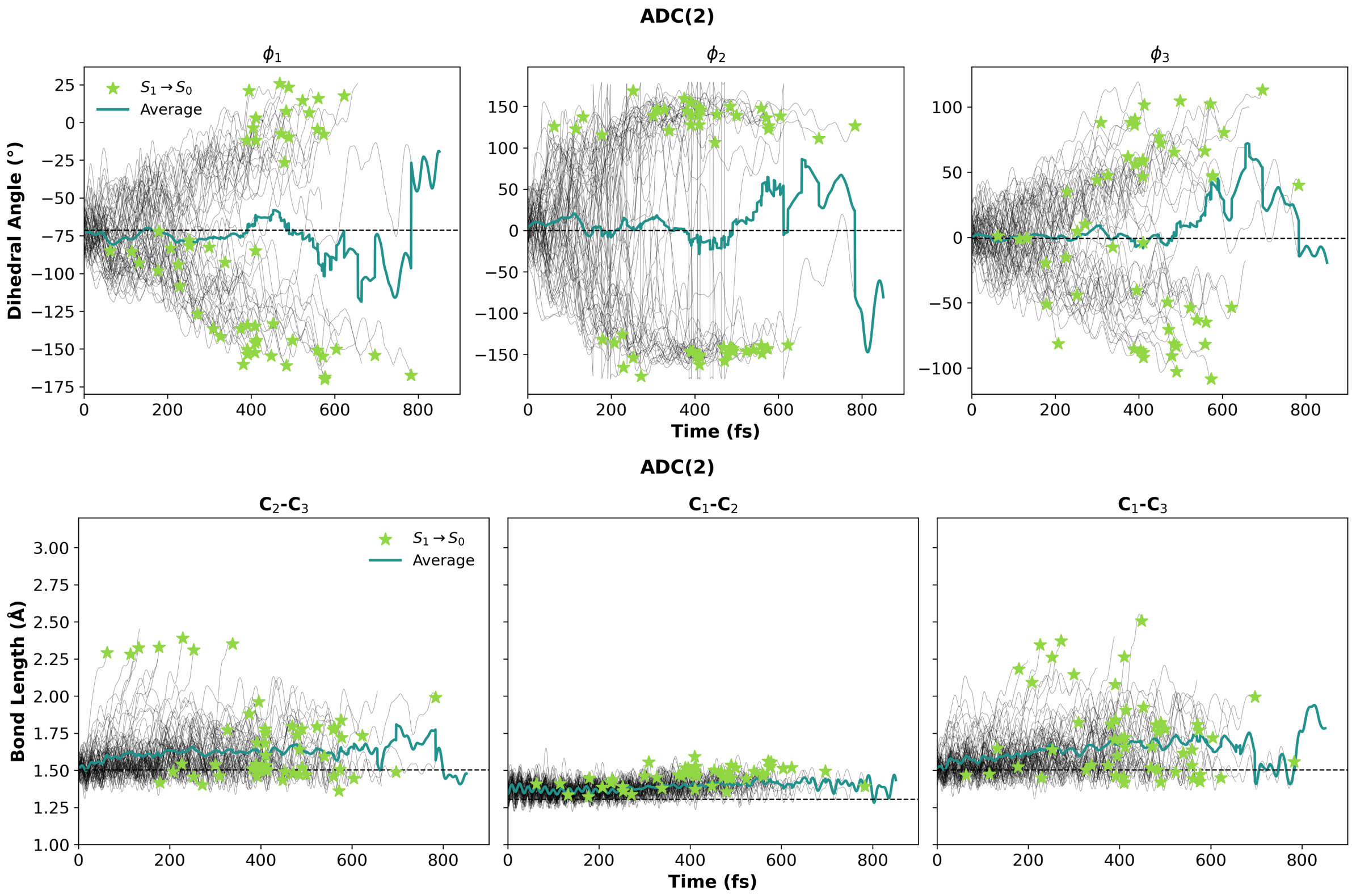

Figure S11: Reaction coordinates for all trajectories, with the respective hops for ADC(2) based dynamics

S13 Hopping Data

Figure S12 shows the cumulative S_1 to S_0 hops over time. A trend similar to that observed for the state populations is evident, with MRSF-TD-CAM-B3LYP lying between the faster ADC(2) dynamics and the slower LR-TD-CAM-B3LYP results.

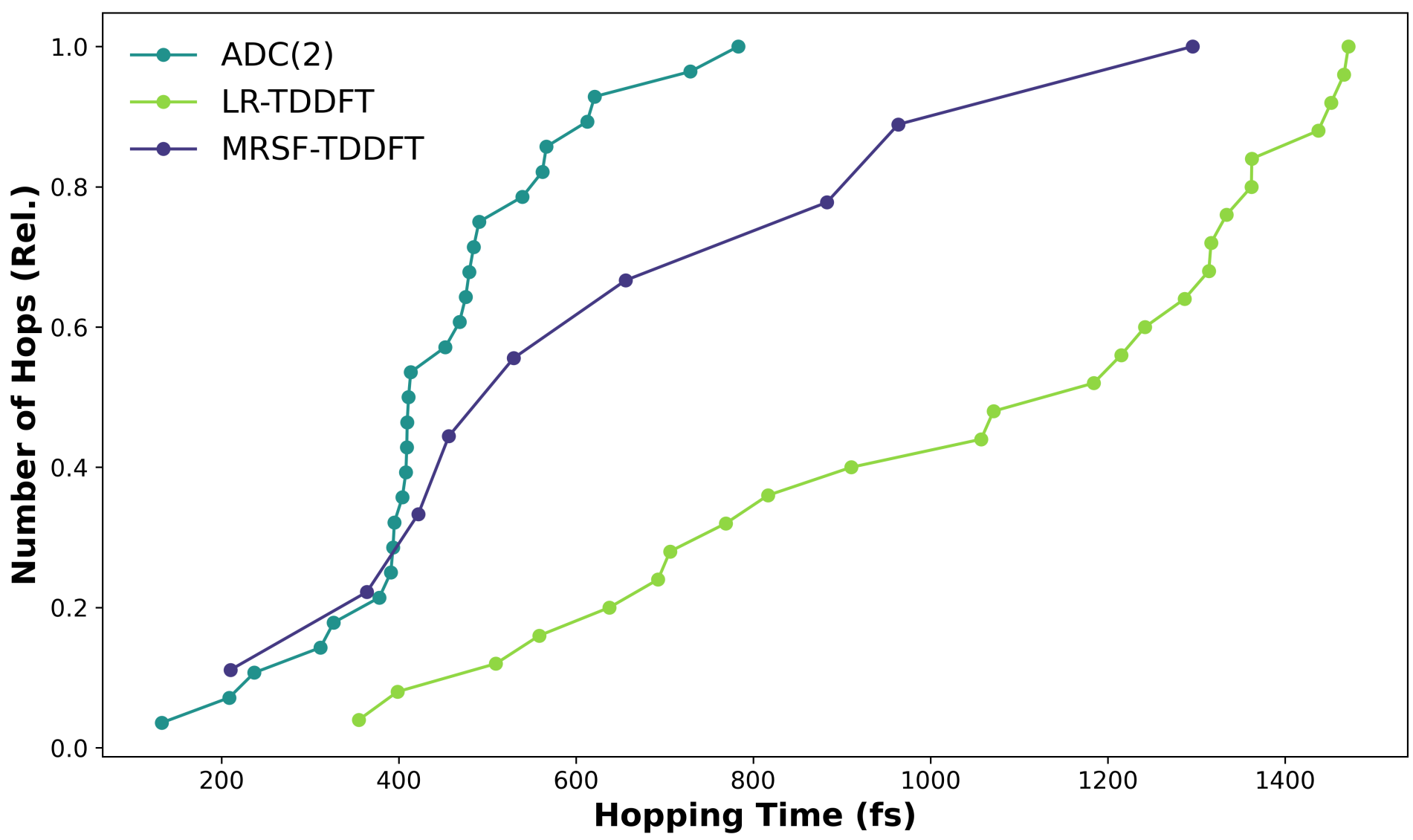

Figure S12: The amount of hops to the ground state overtime with respect to time of the dynamics.

S14 Electronic Population Convergence Test

To test the convergence of the electronic populations, we calculate the the mean absolute deviation at 50%, 60%, 80%, and 100% of the set of trajectories, randomly subsampling 10 times for each fraction.

$$\mathrm{MAD}_f^{(k)} = \frac{1}{N_t} \sum_{i=1}^{N_t} \left| P_f^{(k)}(t_i) - P_{100\%}(t_i) \right| \tag{S13}$$

$$\langle \mathrm{MAD}_f \rangle = \frac{1}{N_{\mathrm{draws}}} \sum_{k=1}^{N_{\mathrm{draws}}} \mathrm{MAD}_f^{(k)} \tag{S14}$$

where $P_f^{(k)}(t_i)$ is the (smoothed, normalised) mean state population at time t_i for the k-th random draw of trajectories at fraction f, $P_{100\%}(t_i)$ is the corresponding population from the full ensemble, N_t is the number of time points, and $N_{\mathrm{draws}} = 10$.

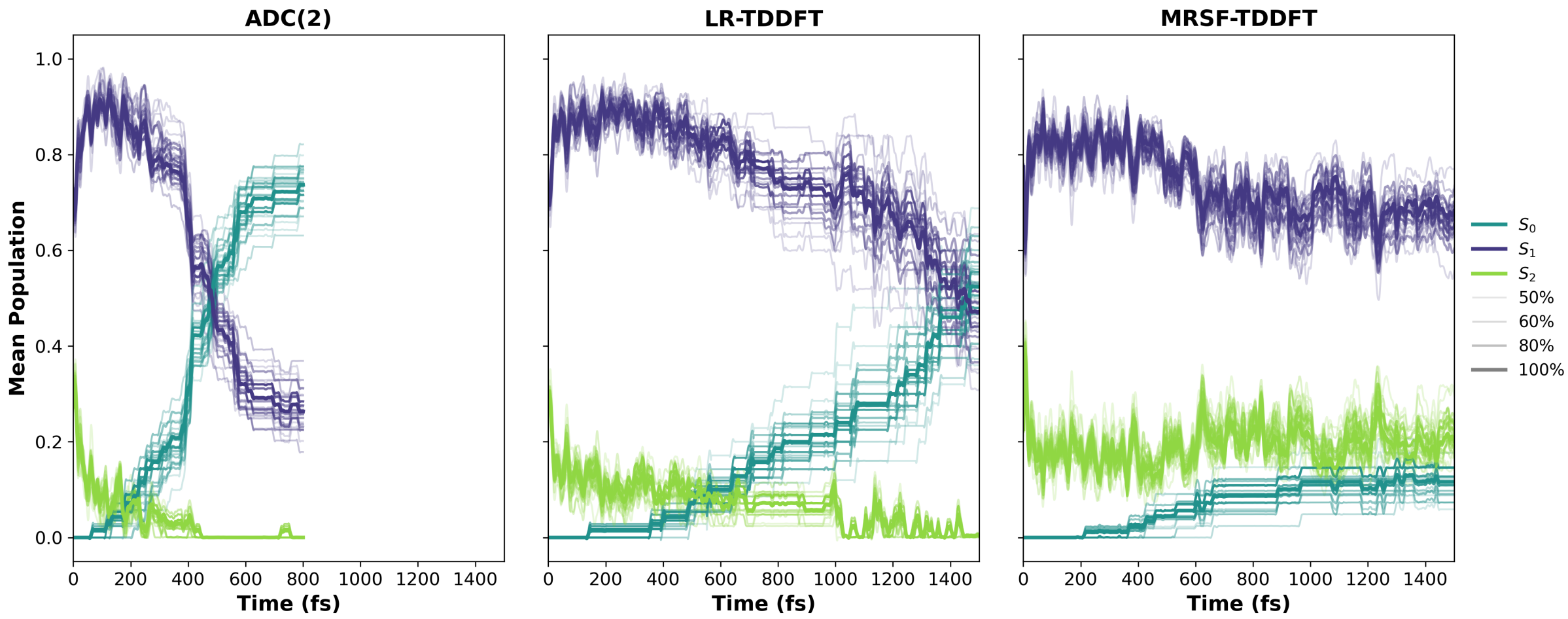

Figure S13: Convergence test of the electronic populations.

Table S8: Mean absolute deviation (MAD) of state populations relative to the full trajectory ensemble (100%), computed for randomly sampled subsets containing 50%, 60%, and 80% of trajectories ($N_{\text{draws}} = 10$).

	**50%**			**60%**			**80%**		
	S_0	S_1	S_2	S_0	S_1	S_2	S_0	S_1	S_2
**ADC(2)**	0.0314	0.0380	0.0119	0.0300	0.0304	0.0095	0.0207	0.0217	0.0054
**LR-TDDFT**	0.0541	0.0597	0.0143	0.0283	0.0324	0.0137	0.0275	0.0294	0.0078
**MRSF-TDDFT**	0.0231	0.0374	0.0331	0.0220	0.0240	0.0247	0.0130	0.0230	0.0175

For all three methods, $\langle \text{MAD}_{80\%} \rangle$ remains below 0.028 for S_0 and S_1, and below 0.018 for S_2, indicating that the trajectory ensembles are well converged. Supplemented by the visual representation in Fig. S13 by the general trend of convergence to the 100% fraction of trajectories when increasing the percentage.

S15 Example MRSF character

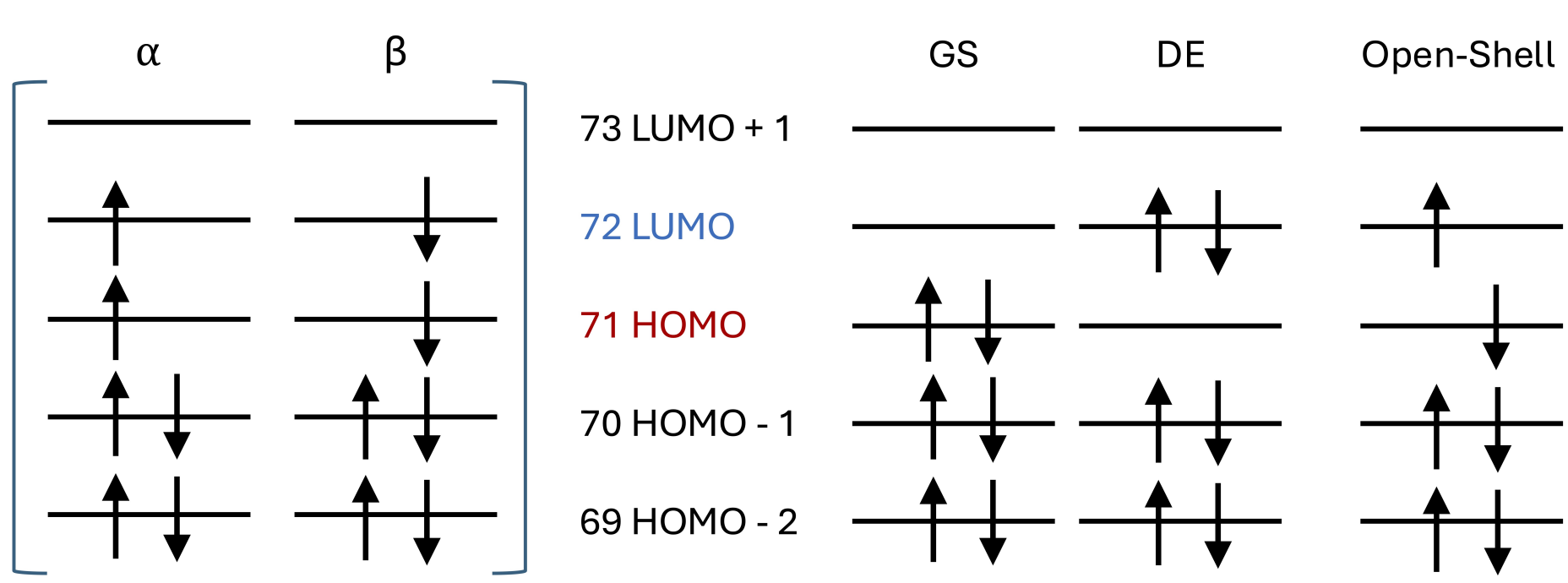

Figure S14: Example configurations for MRSF-TDDFT.

S16 ωB97XD-4 NAMD Trajectories

We include the key reaction coordinates as a function of time in Fig. S15. Additionally, we present two example trajectories in Fig. S16 and Fig. S17.

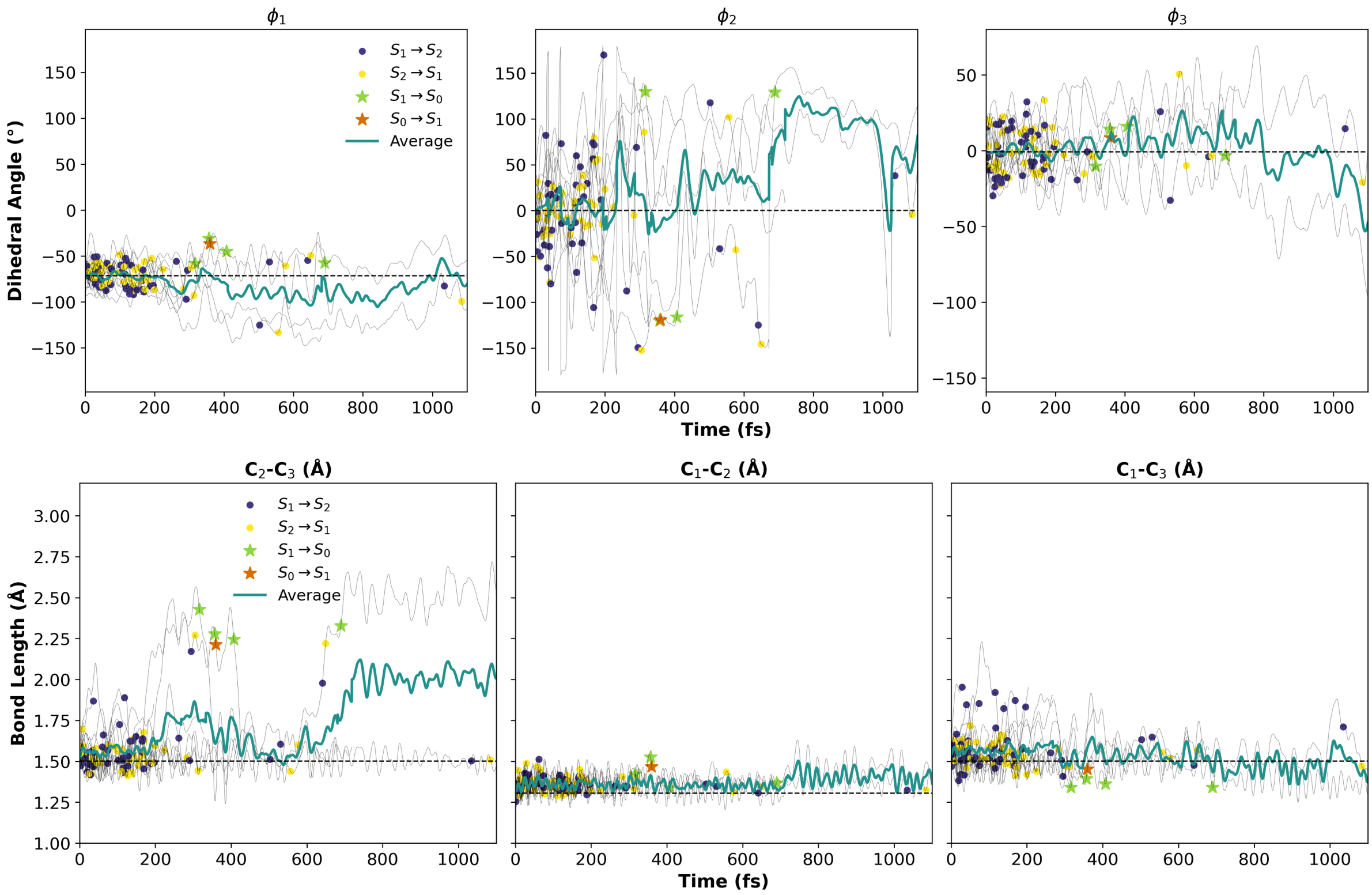

Figure S15: Reaction Coordinates for ωB97XD dynamics, subset of 20 trajectories.

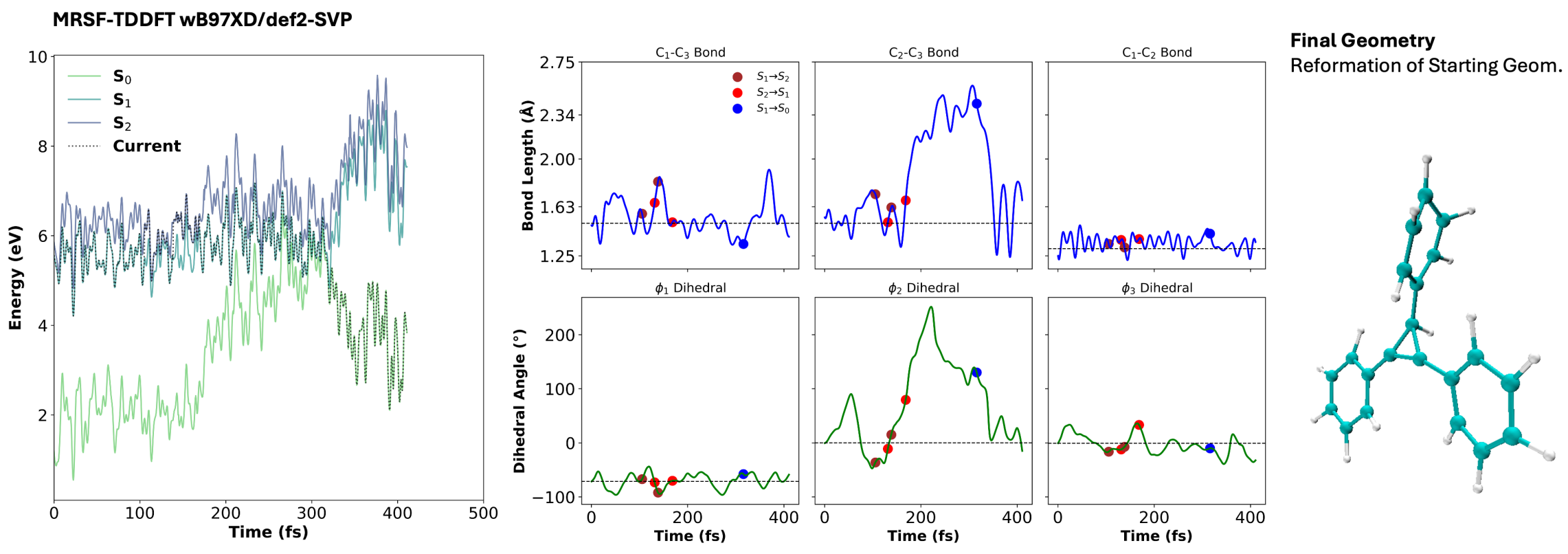

Figure S16: Example trajectory from ωB97XD dynamics, showing the ground state hop leading to the formation of the starting geometry.

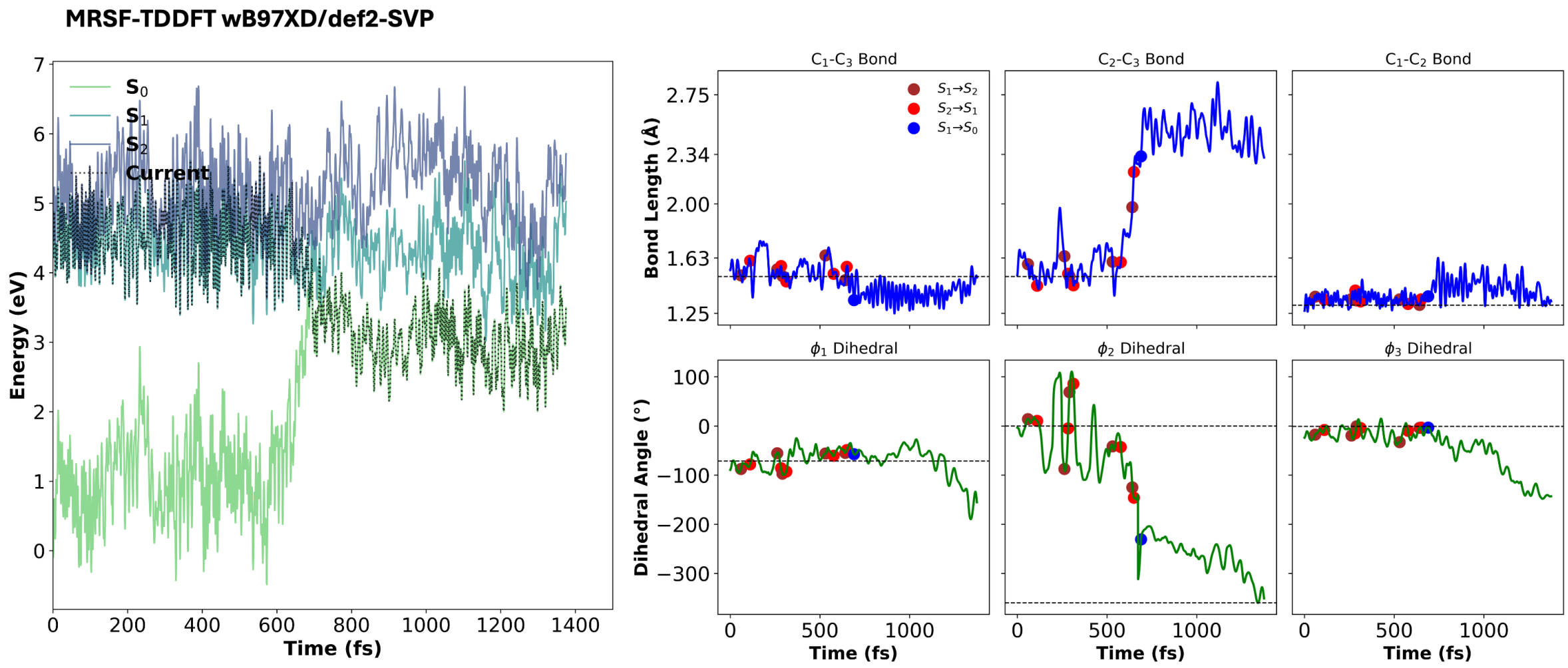

Figure S17: Example trajectory from ωB97XD dynamics

References

[1] David R. Yarkony. On the adiabatic to diabatic states transformation near intersections of conical intersections. *The Journal of Chemical Physics*, 112(5):2111–2120, 02 2000.

[2] David R. Yarkony. Conical intersections: the new conventional wisdom. *The Journal of Physical Chemistry A*, 105(26):6277–6293, 2001.

[3] Ignacio Fdez. Galván, Mickaël G. Delcey, Thomas Bondo Pedersen, Francesco Aquilante, and Roland Lindh. Analytical state-average complete-active-space self-consistent field nonadiabatic coupling vectors: Implementation with density-fitted two-electron integrals and application to conical intersections. *Journal of Chemical Theory and Computation*, 12(8):3636–3653, 2016.

[4] Gregory J. Atchity, Sotirios S. Xantheas, and Klaus Ruedenberg. Potential energy surfaces near intersections. *The Journal of Chemical Physics*, 95(3):1862–1876, 08 1991.

[5] Mario Barbatti, Mattia Bondanza, Rachel Crespo-Otero, Baptiste Demoulin, Pavlo O. Dral, Giovanni Granucci, Fábris Kossoski, Hans Lischka, Benedetta Mennucci, Saikat Mukherjee, Marek Pederzoli, Maurizio Persico, Max Pinheiro Jr, Jiří Pittner, Felix Plasser, Eduarda Sangiogo Gil, and Ljiljana Stojanovic. Newton-x platform: New software developments for surface hopping and nuclear ensembles. *Journal of Chemical Theory and Computation*, 18(11):6851–6865, 2022. PMID: 36194696.

[6] Jan-Robert Vogt, Michael Schulz, Rafael Souza Mattos, Mario Barbatti, Maurizio Persico, Giovanni Granucci, Jürg Hutter, and Anna Hehn. A density functional theory and semiempirical framework for trajectory surface hopping on extended systems. *Journal of Chemical Theory and Computation*, 21(20):10474–10488, 2025.

[7] Jiri Sturala, Marc K. Etherington, Aisha N. Bismillah, Heather F. Higginbotham, William Trewby, Juan A. Aguilar, Elizabeth H. C. Bromley, Alyssa-Jennifer Avestro, Andrew P. Monkman, and Paul R. McGonigal. Excited-state aromatic interactions in the aggregation-induced emission of molecular rotors. *Journal of the American Chemical Society*, 139(49):17882–17889, 2017. PMID: 29151342.

[8] Juliana Cuéllar-Zuquin, Ana Julieta Pepino, Ignacio Fdez. Galván, Ivan Rivalta, Francesco Aquilante, Marco Garavelli, Roland Lindh, and Javier Segarra-Martí. Characterizing conical intersections in dna/rna nucleobases with multiconfigurational wave functions of varying active space size. *Journal of Chemical Theory and Computation*, 19(22):8258–8272, 2023.

[9] M. Brady and R. Crespo-Otero. Is mrsf-tddft suitable for cyclobutanone dynamics? the role of higher energy states in surface hopping simulations. *The Journal of Chemical Physics*, 163(23):234118, 12 2025.

[10] Eya Derbali, Davide Avagliano, Mourad Telmini, and Lea M. Ibele. Robustness and reliability of different caspt2 flavors for nonadiabatic molecular dynamics. *The Journal of Chemical Physics*, 164(14):144104, 04 2026.
